# Biobank-Scale Plasma Proteomics Identifies Novel Biomarkers in Hypertrophic Cardiomyopathy

**DOI:** 10.1161/CIRCGEN.125.005325

**Published:** 2026-04-22

**Authors:** Jonathan H. Chan, Christopher Grace, Mohsen Mazidi, Robert Clarke, Carolyn Y. Ho, Stefan Neubauer, Christopher M. Kramer, Hugh Watkins, Anuj Goel, on behalf of HCMR Investigators, William Weintraub

**Affiliations:** Cleveland Clinic; Oxford University, UK; Oxford University, UK; Yale; Yale; Berlin – Charite, Germany; Calgary, Canada; London - Kings College, UK; University of Michigan; Northwestern University; Erasmus, the Netherlands; Birmingham, UK; UVA Health System; UVA Health System; OHSU; OHSU; London – Royal Brompton, UK; London - Chest Hospital, UK; Leeds, UK; Tufts Medical Center; Tufts Medical Center; Stuttgart, Germany; Bristol, UK; Bristol, UK; Cornell; Duke; Leicester, UK; Amsterdam, the Netherlands; Amsterdam, the Netherlands; Mayo Clinic; Mayo Clinic; Southampton, UK; Aberdeen, UK; Montréal Heart Institute, Canada; Florence, Italy; University of Toronto, Canada; University of Toronto, Canada; University of Pennsylvania; London - St. Georges, UK; London - St. Georges, UK; Bologna, Italy; Edinburgh, UK; Heidelberg, Germany; Glasgow, UK; St. Luke’s Mt. Sinai; Quebec City, Canada; Johns Hopkins University; Johns Hopkins University; New York University; Beth Israel Deaconess; Beth Israel Deaconess; Methodist Hospital, Houston; Milan, Italy; McGill University, Canada; McGill University, Canada; Rome Sapienza, Italy; 1 Division of Cardiovascular Medicine, Radcliffe Department of Medicine (J.H.C., C.G., S.N., H.W., A.G.), University of Oxford, United Kingdom.; 2 Centre for Human Genetics, Nuffield Department of Medicine (J.H.C., C.G., H.W., A.G.), University of Oxford, United Kingdom.; 3 Nuffield Department of Population Health (M.M., R.C.), University of Oxford, United Kingdom.; 4 Cardiovascular Division, Department of Medicine and Department of Radiology, Brigham and Women’s Hospital, Boston, MA (C.Y.H.).; 5 Cardiovascular Division, University of Virginia Health System, Charlottesville, VA (C.M.K.).

**Keywords:** biomarkers, blood proteins, genetics, humans, prognosis

## Abstract

**BACKGROUND:**

Hypertrophic cardiomyopathy (HCM) is characterized by substantial heterogeneity in both clinical phenotype and risk of adverse outcomes, including heart failure and sudden cardiac death. This highlights the need for robust biomarkers for risk stratification, and while previous studies have identified the role of select plasma proteins, comprehensive large-scale proteomic analyses have been limited in HCM.

**METHODS:**

We performed a case-control analysis of 2922 plasma proteins in 49 588 UK Biobank participants (100 HCM cases) to identify proteins associated with HCM. External replication analyses were performed in the deCODE Genetics Icelandic study (51 cases/38 904 controls) and All of Us (546 cases/41 049 controls) data sets. Associations with adverse clinical outcomes and cardiac endophenotypes of disease severity were further identified, and causal relationships were evaluated using Mendelian randomization. Relative biomarker importance was also assessed by joint modeling via machine learning.

**RESULTS:**

We identified novel associations of ANGPT2 (angiopoietin-2) and LTBP2 (latent transforming growth factor-beta binding protein 2) with HCM, with both also showing prognostic utility for heart failure-related outcomes in HCM cases. We also confirmed the associations of established biomarkers (eg, NT-proBNP [N-terminal pro-B-type natriuretic peptide], troponins I and T) with HCM cases, cardiac imaging markers of disease severity, and adverse outcomes. Mendelian randomization analyses supported a causal effect of HCM on increasing NT-proBNP and troponin T levels.

**CONCLUSIONS:**

This biobank-scale plasma proteomic study in HCM identified ANGPT2 and LTBP2 as novel HCM biomarkers with potential diagnostic and prognostic utility. These findings highlight the potential for plasma proteomics to improve risk prediction and provide insight into HCM pathobiology.

Hypertrophic cardiomyopathy (HCM) is one of the most common inherited heart diseases, afflicting 1 in 500 people worldwide.^1^ It is characterized by hypertrophy of the left ventricle which can cause left ventricular dysfunction, heart failure (HF), and sudden cardiac death.^2^ This potential severity in conjunction with its marked clinical heterogeneity^2^ have prompted the need for reliable biomarkers to aid risk stratification. Plasma proteins present an accessible, low-cost modality to evaluate underlying pathological changes and have aided risk prediction for various diseases.^3^ In HCM, prior studies have implicated proteins including NT-proBNP (N-terminal pro-B-type natriuretic peptide) and troponins, which reflect pathophysiological processes such as myocardial wall stress and necrosis.^4^ Various cohort studies have also demonstrated their associations with imaging-derived endophenotypes of disease severity, including myocardial fibrosis,^5,6^ and with adverse clinical outcomes.^7,8^

The advent of biobank-scale plasma proteomic data sets, such as the UK Biobank Pharma Proteomics Project (UKB-PPP) measuring ≈3000 plasma proteins in >50 000 individuals,^9^ has enabled comprehensive, population-scale analyses. These have identified novel biomarkers and disease mechanisms in cardiac conditions such as HF, myocardial infarction, and coronary artery disease.^3,10^ In this study (Figure 1), we leveraged the UKB-PPP to evaluate the association of 2922 proteins with HCM to discover disease-associated plasma protein biomarkers. We further validated a subset of these biomarkers in external replication data sets, including the deCODE Genetics SomaScan V4 study, All of Us (AoU), and HCMR cohorts (HCM Registry), and demonstrated their prognostic utility through association with adverse clinical outcomes. We also explored their causal relevance for HCM using Mendelian randomization and leveraged machine learning-based classifiers to infer their relative importance in a joint setting. Together, these analyses refine our understanding of the plasma proteomic landscape in HCM, delineating the diagnostic and prognostic utility of both established and novel biomarkers.

**Figure 1.**
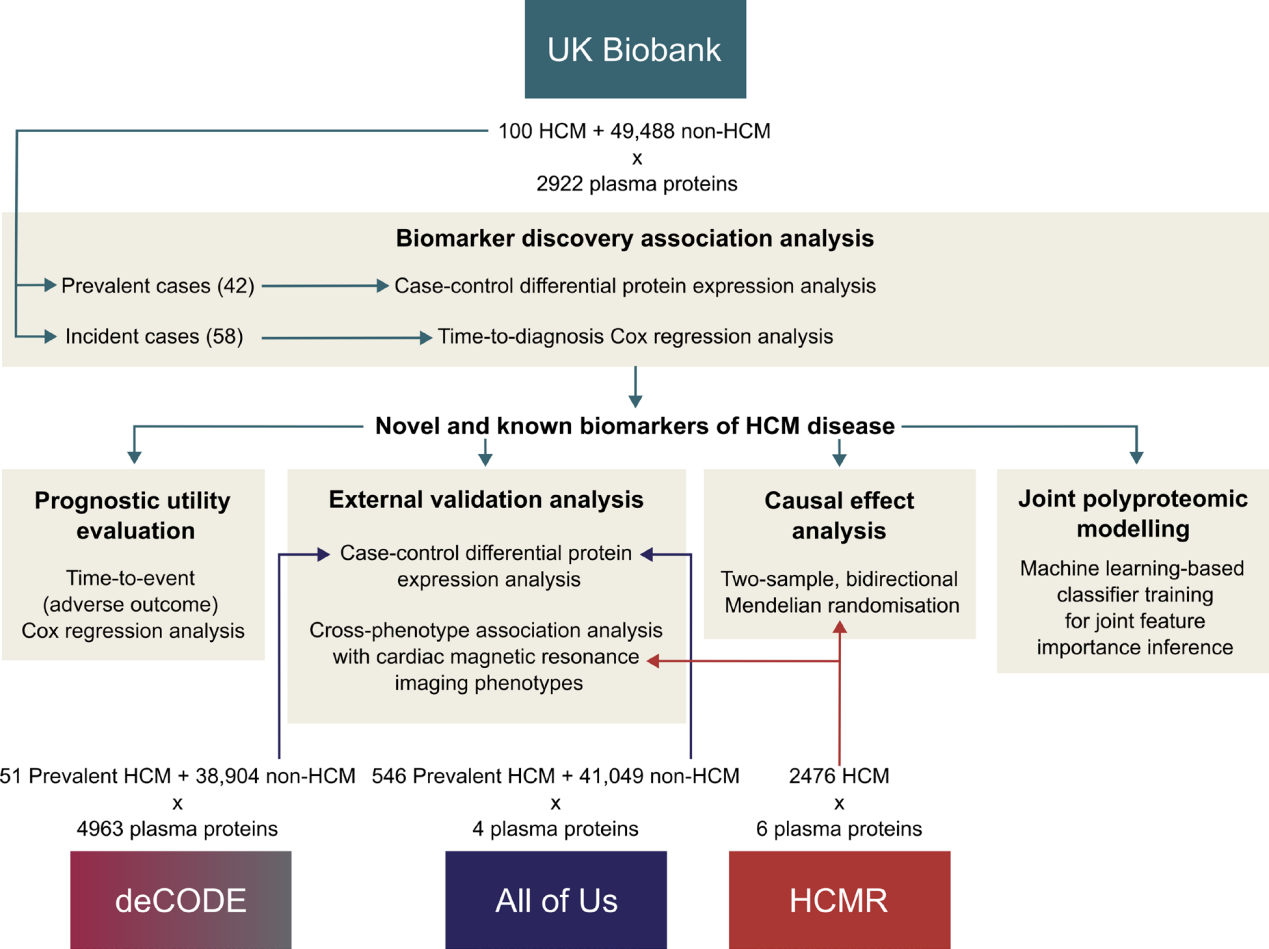
**Overview of analyses and data sets involved.** Hypertrophic cardiomyopathy (HCM) cases in UK Biobank, deCODE Genetics, and All of Us data sets were divided into prevalent cases (those diagnosed before the blood sample collection date or within 5 years) and incident cases (those diagnosed after the blood sample collection date +5 years). This represents an assumed potential lag between disease manifestation and clinical diagnosis. HCMR indicates Hypertrophic Cardiomyopathy Registry.

## Methods

Full methods detailed in the Supplemental Material. Analysis code is provided at https://github.com/JonChan0/HCM_Plasma_Proteomics. UK Biobank (UKB) and AoU data available to approved researchers at https://biobank.ndph.ox.ac.uk/showcase/ and https://www.researchallofus.org/. The National Research Ethics Service approved the UKB (16/NW/0274) and the HCMR studies (14/SC/0190). The National Bioethics Committee of Iceland approved the deCODE study. All patients provided written informed consent.

## Results

### Discovery Association Analysis Identified Known and Novel Biomarkers of HCM

**Figure 2.**
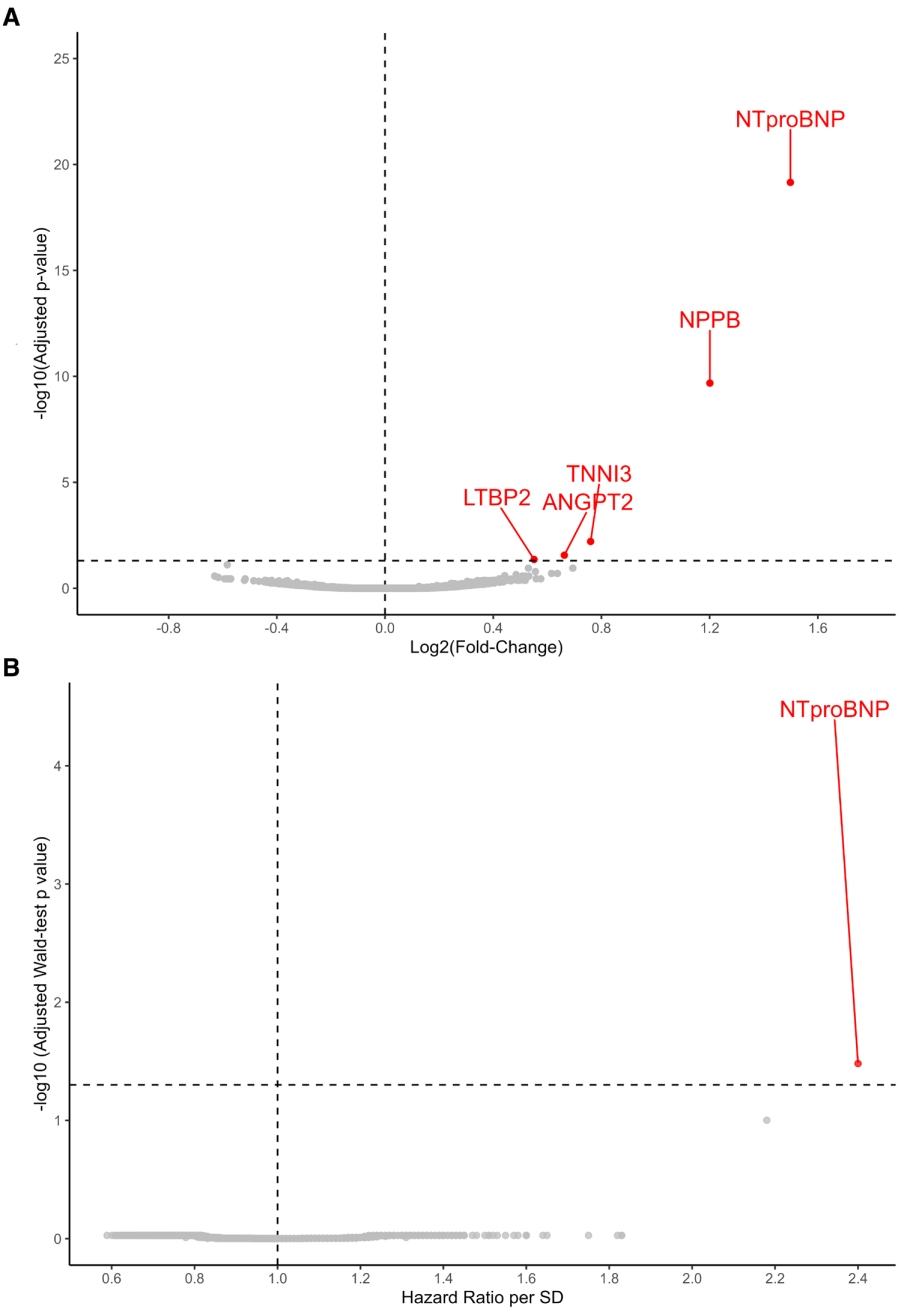
**Plasma proteomic association analyses in UK Biobank identified known and novel hypertrophic cardiomyopathy (HCM) biomarkers. A**, Case-control differential protein expression analysis using prevalent HCM cases identified previously reported biomarkers of disease (eg, NT-proBNP [N-terminal pro-B-type natriuretic peptide]) and novel ones (ANGPT2 [angiopoietin-2] and LTBP2 [latent transforming growth factor-beta binding protein 2]). **B**, Time-to-event analysis of incident HCM diagnosis in the controls from above analysis (which includes incident HCM cases) provided orthogonal support for NT-proBNP’s association with disease. Multiple testing correction (MTC) applied via Benjamini-Hochberg procedure to control false discovery rate at 5%.

The UKB-PPP (Table S1) enabled discovery association analysis across 2922 plasma proteins in 49 588 individuals (100 HCM cases). Our case-control differential expression analysis identified 5 proteins significantly associated with prevalent HCM (Figure 2A). These included established biomarkers such as NT-proBNP, its related NPPB (brain natriuretic peptide), and TNNI3 (troponin I).^4^ The remaining 2 biomarkers, ANGPT2 (angiopoietin-2) and LTBP2 (latent transforming growth factor-beta binding protein 2), had not previously been associated with HCM disease status. Among the 49 546 controls (including 58 incident HCM cases), we performed time-to-event analysis to evaluate associations between baseline protein levels and incident HCM diagnosis in UKB-PPP, with a median follow-up time of 14.9 years (interquartile range, 14.1–15.7 years). This provided orthogonal support for NT-proBNP (hazard ratio (HR) per SD, 2.40 [95% CI, 1.81–3.19], *P*=1.13×10^−5^; Figure 2B).

Some of the HCM-associated biomarkers had previously been associated with HF,^11^ so we repeated case-control analyses in the non-HCM HF cohort (Figure S1A). All HCM biomarkers except troponin I were also associated with HF, indicating that they likely reflect pathobiological changes, including cardiac wall stress and cellular damage, which are known features of all-cause HF. However, the overall signal in HCM was not driven solely by HF-positive HCM cases, because analysis of non-HF HCM cases showed significant associations with 3 of these 5 biomarkers (Figure S1B).

### Biomarkers Were Validated in External deCODE Genetics, AoU, and HCM Registry Data Sets

**Figure 3.**
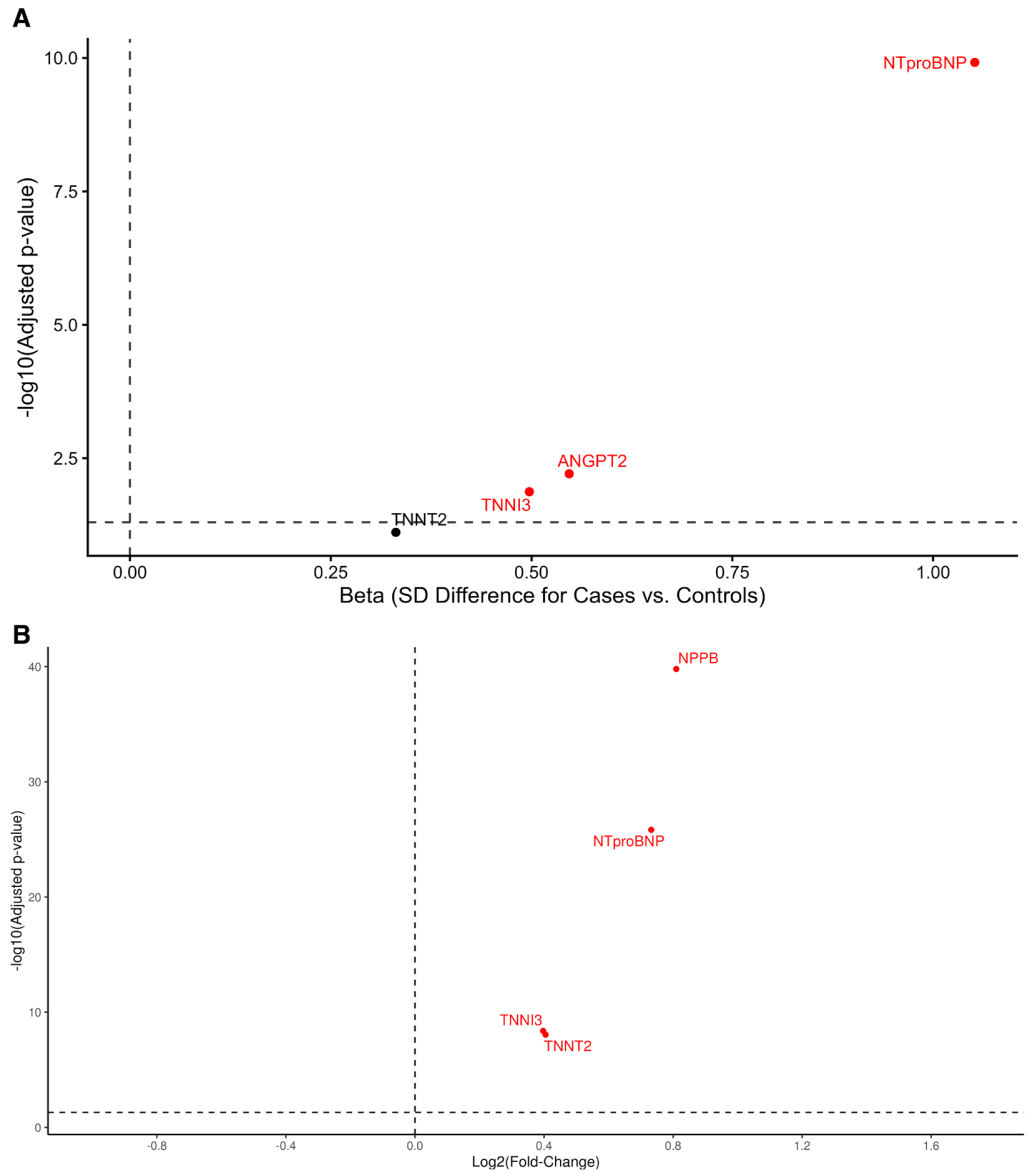
**Known and novel biomarkers were validated in external deCODE Genetics and All of Us data sets.** Case-control differential protein expression analysis in external replication (**A**) DeCODE Genetics and (**B**) All of Us data sets validated known hypertrophic cardiomyopathy (HCM) associations of biomarkers NT-proBNP (N-terminal pro-B-type natriuretic peptide) and Troponin I (TNNI3), and novel biomarker ANGPT2 (angiopoietin-2). Plasma proteins tested in external replication deCODE Genetics data set included all those significant at the 5% FDR threshold (Figure 2A) if they were also measured in the SomaScan V4 study. Multiple testing correction (MTC) applied via Benjamini-Hochberg procedure to control false discovery rate at 5% (dashed horizontal line).

The deCODE Genetics SomaScan V4 study provided Icelandic population-scale data for external replication of significant findings from the primary analysis. Of the 5 HCM-associated biomarkers, both known (NT-proBNP and troponin I) and novel (ANGPT2) biomarkers were replicated in this external study, with the other 2 (NPPB and LTBP2) not tested due to data unavailability (Figure 3A). This highlights the robustness of these signals, especially of the novel biomarker ANGPT2, across 2 distinct population cohorts in the United Kingdom and Iceland.

The AoU cohort (Table S2) also provided population-scale data for known biomarkers NT-proBNP, NPPB, and troponins I and T. Such proteins replicated, albeit with smaller effect sizes (Figure 3B). This may reflect AoU’s nonstandardized measurements and differences in cohort composition relative to UKB. Sensitivity analysis of the HF-only subgroup replicated the biomarker nonspecificity observed in UKB (Figure S1C).

HCMR provided a case-only data set with joint cardiac magnetic resonance imaging and plasma protein data (Table S3). As such, we also investigated cross-phenotype correlations between plasma proteins and cardiac magnetic resonance imaging-derived left ventricular measures in HCMR. We specifically evaluated measures of hypertrophy, fibrosis, and contractile function. Increased levels of NT-proBNP and troponin T were associated with greater hypertrophy, fibrosis, and less contractile function (Table). These external data sets ultimately supported both novel associations such as ANGPT2, and known associations of NT-proBNP/troponins with HCM,^4^ and also provided insight into their link to imaging-derived endophenotypes of severity.

**Table.**
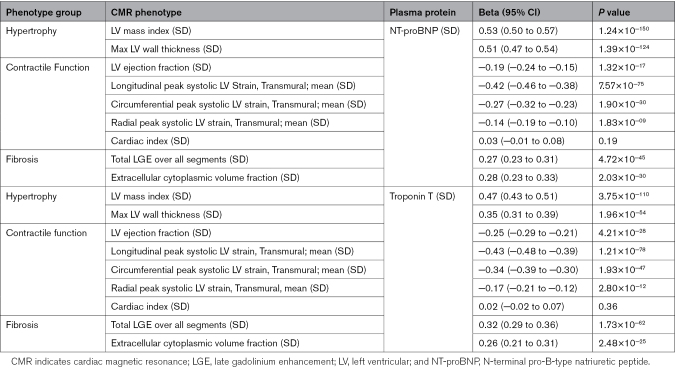
Cross-Phenotype Association Analyses in Hypertrophic Cardiomyopathy Registry of NT-proBNP and Troponin T With Imaging-Derived Measures of Hypertrophy, Fibrosis, and Contractile Function

### Known (NT-proBNP) and Novel Biomarkers (ANGPT2 and LTBP2) Showed Prognostic Utility in UKB Cases

**Figure 4.**
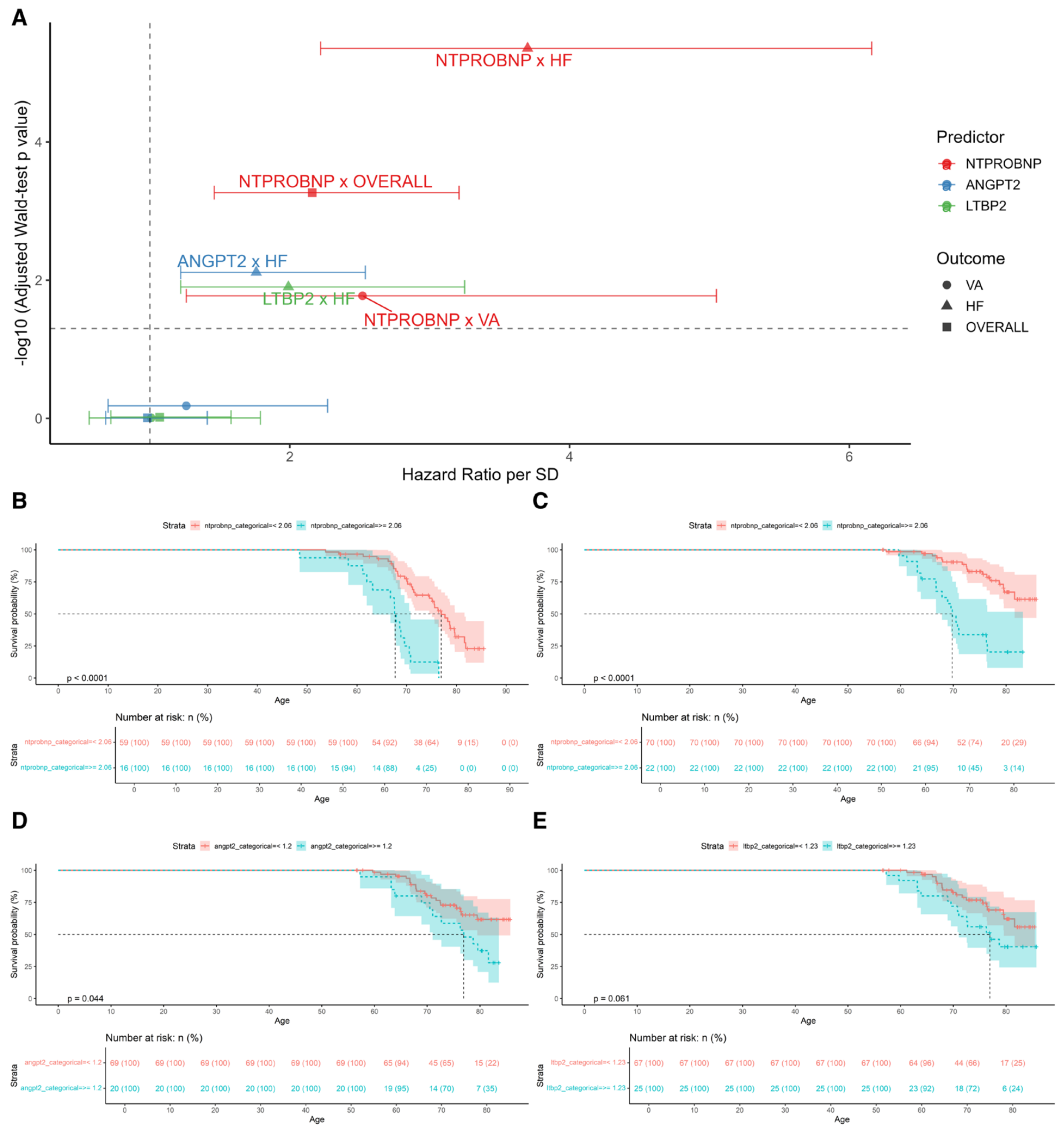
**Known and novel biomarkers showed potential prognostic utility. A**, Multivariable Cox regression with incident adverse clinical outcomes in UK Biobank showed associations of known (NT-proBNP [N-terminal pro-B-type natriuretic peptide]) and novel (ANGPT2 (angiopoietin-2) and LTBP2 [latent transforming growth factor-beta binding protein 2]) biomarkers with composite outcomes after covariate adjustment. Error bars indicate 95% CIs. Multiple testing correction applied via Benjamini-Hochberg procedure to control false discovery rate at 5%. **B** through **E**, Kaplan-Meier event curves with stratification by (**B** and **C**) NT-proBNP (with overall and heart failure [HF] composites, respectively), (**D**) ANGPT2 and (**E**) LTBP2 levels (both with HF composite) at baseline (threshold at 75th percentile: 2.06, 1.2, and 1.23 SD, respectively) showed significant difference in event incidence between <75th percentile and ≥75th percentile subgroups for biomarker-composite combinations before covariate adjustment. Statistical test for difference in subgroups via log-rank test. VA indicates ventricular arrhythmia.

Given these indirect associations with disease severity, we also directly investigated the association of known (NT-proBNP) and novel (ANGPT2 and LTBP2) biomarkers with adverse clinical outcomes in UKB cases. Median follow-up times (years) for each of the composite outcomes were 11.2 (interquartile range, 7.5–14.5) for Overall, 14.3 (interquartile range, 9.7–15.4) for HF, and 14.7 (13.9–15.5) for ventricular arrhythmia, with summary numbers of events detailed in Table S4. Consistent with previous work,^8^ NT-proBNP was a significant predictor of adverse clinical outcomes including the Overall (HR per SD, 2.16 [95% CI, 1.46–3.21]; *P*=1.0×10^−4^), HF (HR per SD, 3.70 [95% CI, 2.22–6.16]; *P*=4.91×10^−7^), and ventricular arrhythmia (HR per SD, 2.52 [95% CI, 1.16–5.05]; *P*=0.0093) composites. ANGPT2 and LTBP2 were also associated with incidence of the HF composite (HR per SD, 1.76 [95% CI, 1.22–2.54]; *P*=0.0026) and (HR per SD, 1.99 [95% CI, 1.22–3.25]; *P*=0.0056), respectively (Figure 4A). Stratification of cases by their plasma protein levels further supported these associations (Figure 4B through 4D). These time-to-event analyses validated NT-proBNP’s prognostic utility and identified ANGPT2 and LTBP2 as novel biomarkers associated with HF-related outcomes in patients with HCM.

### Mendelian Randomization Analyses Established Causal Relationships Between HCM and Its Biomarkers NT-proBNP and Troponin T

The analyses so far represent observational approaches without clarification of underlying causality. Thus, we also evaluated the causal relevance of these associations using Mendelian randomization. We used common genetic variants associated with phenotypes as instrumental variables to test whether differences in genetically determined levels of an exposure were associated with an outcome independently of unmeasured confounders.^12^

**Figure 5.**
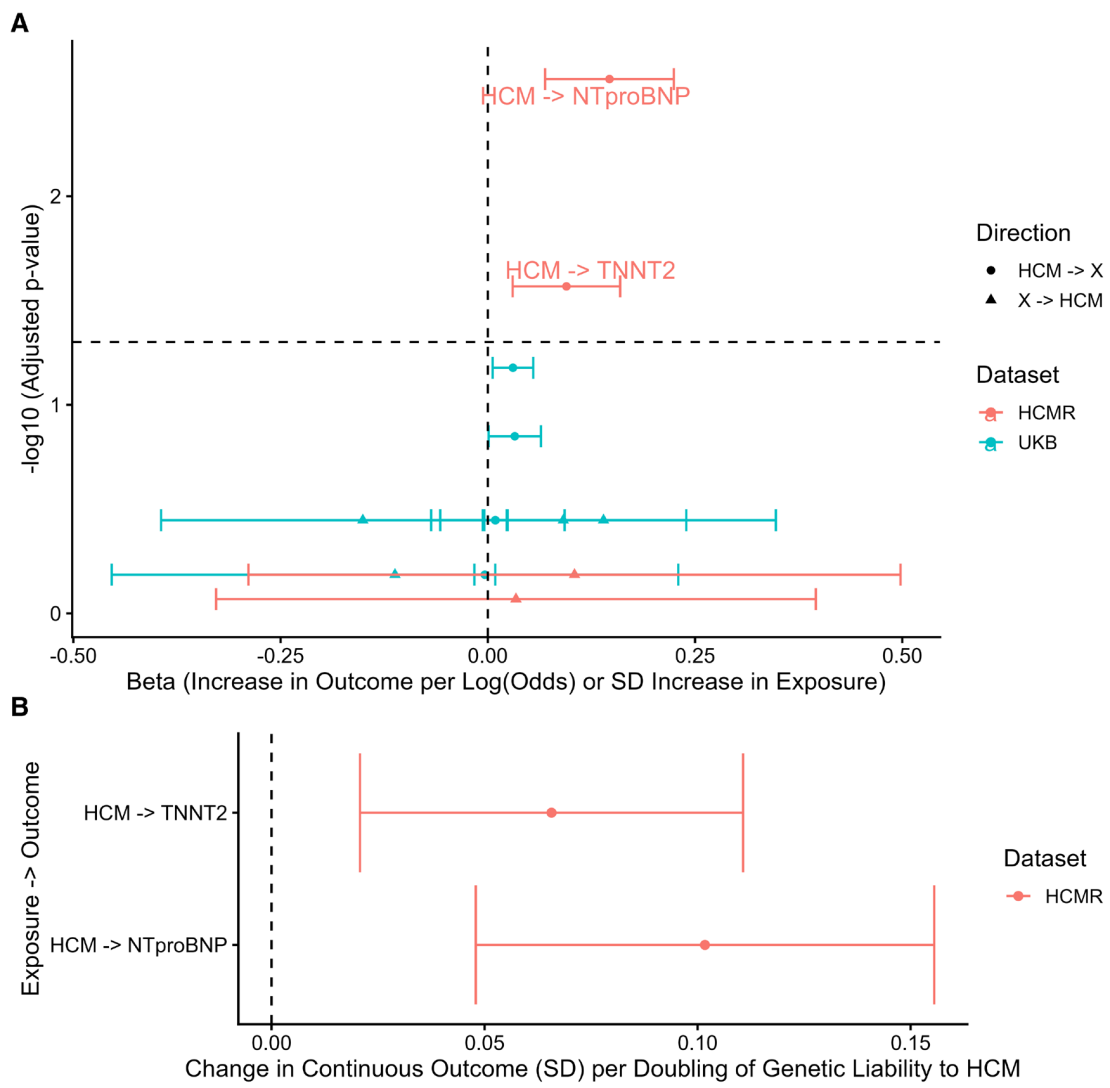
**Mendelian randomization analyses established causal associations between hypertrophic cardiomyopathy (HCM) and known biomarkers. A**, Bidirectional, 2-sample Mendelian randomization analyses showed significant causality between HCM disease status and plasma proteins (X) prioritized by case-control association analyses. Multiple testing correction (MTC) applied via Benjamini-Hochberg procedure to control false discovery rate at 5%. Associations labeled if significant after MTC. **B**, Forest plot of significant conclusions indicated that common genetic risk of HCM causes increased NT-proBNP (N-terminal pro-B-type natriuretic peptide) and Troponin T (TNNT2) levels. Error bars represent 95% CIs. HCMR indicates Hypertrophic Cardiomyopathy Registry; and UKB, UK Biobank.

Analyses demonstrated that increased genetic liability to HCM was causally associated with higher plasma levels of NT-proBNP and troponin T levels with effects of 0.10 (95% CI, 0.05–0.16) and 0.07 (95% CI, 0.02–0.11) standard deviations, respectively, per doubling of liability (Figure 5). Sensitivity analyses (Figures S4 and S5), including colocalization analyses (Figure S6) highlighted the importance of variants at *BAG3* and *MYOZ1/SYNPO2L* loci, respectively, in driving effects, but these analyses were constrained by the limited power of the HCMR-derived genome-wide association studies (GWAS). From the UKB-derived GWASs of plasma proteins, nominally significant signals were obtained for HCM causing increased levels of NT-proBNP and NPPB (Table S5), but these were nonsignificant after multiple testing correction. The discrepancy for the HCM-to-NT-proBNP signal between the HCMR and UKB-derived GWASs may arise from the UKB’s healthy volunteer bias^13^ such that, for UKB’s healthier HCM cases, this may result in lower biomarker levels than the clinically enrolled HCMR cases. As such, the HCM instruments had weaker NT-proBNP associations in the UKB GWAS relative to HCMR’s (Figure S7). Overall, Mendelian randomization showed that HCM causes elevated NT-proBNP and troponin T plasma levels, validating their roles as biomarkers.

### Machine Learning Enabled Joint Proteomic Modeling to Evaluate Relative Predictive Importance

**Figure 6.**
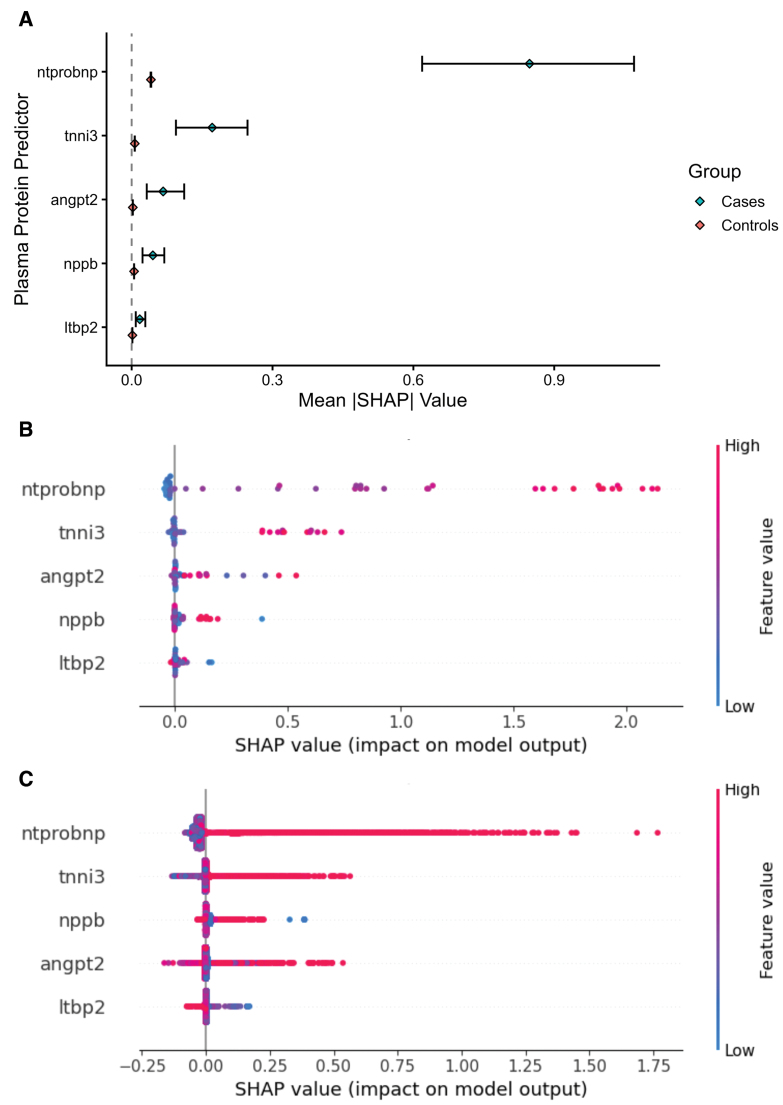
**Machine learning-based joint modeling of prioritized plasma proteins highlighted additive predictive contributions of known and novel biomarkers. A**, Shapley additive explanation (SHAP) values quantify how much a specific protein’s measured level pushed the model’s prediction for an individual toward or away from a case prediction. Mean absolute SHAP values (unit=log[odds of hypertrophic cardiomyopathy]) for each plasma protein for prevalent hypertrophic cardiomyopathy (HCM) cases and controls showed the relative feature importance and impact on prediction by trained XGBoost classifier. NT-proBNP (N-terminal pro-B-type natriuretic peptide) has the greatest predictive contribution in both cases and controls. Error bars represent 95% CIs computed by bootstrapping the evaluated data set. **B** and **C**, Beeswarm plots of SHAP values for each plasma protein stratified for (**B**) cases and (**C**) controls indicates the distribution of SHAP values over all per-individual predictions in the data set. The extended positive tail for NT-proBNP reflects the strong impact of high NT-proBNP values for predicting cases whereas its truncated negative tail reflects a weak impact of low NT-proBNP values in predicting controls. TNNI3 indicates Troponin I; and XGBoost, Extreme-Gradient boosted trees.

Discovery analyses evaluated the relationship between proteins and HCM marginally, with each protein assessed individually. To evaluate these relationships in a joint analysis, we used machine learning to model proteins jointly, capture potential nonlinear effects, and ultimately assess each biomarker’s relative importance. Shapley additive explanation values enabled this by quantifying how much a specific protein’s measured level shifts the model’s prediction for an individual toward or away from case prediction.^14^ As expected, the biomarkers had greater average predictive contribution in cases relative to controls (Figure 6A). Specifically, NT-proBNP had the greatest mean |Shapley additive explanation| value in both groups (cases: 0.85 [95% CI, 0.62–1.07]; controls: 0.041 [95% CI, 0.040–0.042]), reflecting its greatest predictive utility in a combined setting. This was mostly driven by high NT-proBNP values pushing the model toward case prediction (high positive Shapley additive explanation value; Figure 6B and 6C; Figure S8). Troponin I and ANGPT2 also had significant joint contributions (Figure 6A). Medium-high levels pushed individuals toward case prediction, albeit with reduced magnitudes relative to NT-proBNP (Figure 6B and 6C; Figure S8). In a jointly modeled setting, elevated levels of these 3 biomarkers (especially NT-proBNP) had substantial additive predictive utility in predicting cases.

## Discussion

HCM is characterized by clinical heterogeneity and the potential for severe adverse outcomes, motivating a need for biomarkers with predictive and prognostic utility.^2^ Leveraging UKB-PPP and external data sets, including deCODE Genetics, AoU, and HCMR, this study presents a biobank-scale interrogation of the plasma proteome in HCM and highlights both established and novel proteins with potential relevance for diagnosis, prognosis and understanding of latent disease pathways.

These novel biomarkers included ANGPT2 and LTBP2. ANGPT2 is a context-dependent regulator of vascular permeability at the endothelium, controlling the balance between stable, and its permeable, destabilized states.^15^ This is mediated by the Tyrosine kinase receptor with Immunoglobulin and *E*GF homology domain 1/2 (TIE1/2). ANGPT2 has been linked to numerous heart conditions. Specifically, elevated plasma ANGPT2 was associated with HF-linked hospitalization in atrial fibrillation patients,^16^ as well as with incidence and severity of HF in cardiovascular disease-free and HF cohorts, respectively.^17^ This likely reflects shared pathobiological mechanisms with HCM such as endothelial stress. Pathological changes in HCM, as hypertrophy and fibrosis may cause strain on the microvasculature, resulting in protective activation of ANGPT2 expression as an agonist of the TIE2 receptor to inhibit vascular leakage.^15^ However, overexpression of ANGPT2 in mouse hearts resulted in increased endothelial destabilization and myocardial fibrosis,^18^ likely via antagonism of TIE2. This context-dependent activity of ANGPT2 confounds our understanding of its role in HCM, but regardless, elevated levels associate with disease in population-scale data sets from both the UK and Iceland.

LTBP2 is a secreted glycoprotein that incorporates and stabilizes extracellular matrix components such as microfibrils.^19^ While it was not present in the deCODE Genetics SomaScan V4 study for external replication, previous studies have identified it as a marker of cardiac fibrosis, with upregulated expression and protein localization in fibrotic regions from HF mouse models and patient-derived tissues.^20^ A rat dilated cardiomyopathy model study further indicated its role in driving fibrosis, as siRNA-mediated knockdown reduced fibrosis.^19^ Plasma LTBP2 levels have also been correlated with myocardial levels and event-free survival from sudden cardiac death in patients with dilated cardiomyopathy.^21^ This known biology of LTBP2, and the increased plasma levels we observed in HCM, suggest increased expression of this extracellular matrix (ECM) remodeling protein, likely by activated cardiac fibroblasts. As such, plasma LTBP2 may serve as a novel biomarker of HCM reflecting active ECM remodeling and fibrosis.

NT-proBNP and troponins I and T are established biomarkers of HCM.^4,22^ NT-proBNP is produced by proteolysis of pro-brain natriuretic peptide released by cardiomyocytes under wall stress.^4^ In contrast, troponins I and T plasma levels reflect extracellular release following myocardial injury,^4^ or even elevated mechanical stress in the absence of injury.^23^ They were associated with imaging markers of disease severity^6^ and adverse clinical outcomes in patients with HCM.^7,8,24^ Our analyses also confirmed their disease association at the population scale and, via Mendelian randomization, demonstrated the causal role of HCM in increasing NT-proBNP and troponin T levels. NT-proBNP’s association with incident disease may also indicate its ability to reflect subclinical, pathological changes before clinical presentation, as suggested by studies of nonhypertrophic individuals carrying causal sarcomeric mutations.^22,25^

We also leveraged machine learning via Shapley additive explanation analysis to move beyond marginal associations and assess the joint predictive contributions of biomarkers. This identified the importance of NT-proBNP in a multimarker context, driven by its elevated levels signifying case status. Troponin I and ANGPT2 also had significant additive predictive contributions to case prediction in a joint setting, suggesting the utility of a multimarker panel whereby each protein contributes distinct information.

A limitation of our analyses was the heterogeneity of data sets, such that not all data sets measured the same proteins of interest. For example, limited proteins were profiled in HCMR and AoU data sets, and some were not measured in UKB (troponin T) and deCODE (LTBP2). However, this deCODE study enabled external validation of most primary findings despite the only modest correlation in protein measurements previously reported between the differing platforms of Olink and SomaScan V4.^26^ The use of targeted panels in UKB and deCODE also likely biases toward those with known disease associations and limits discovery of unknown proteins with potential associations. A key limitation was also the low number of HCM cases in UKB, which limited the statistical power of our biomarker discovery analyses. We also assumed prevalent case status if individuals were diagnosed up to 5 years after blood collection to capture signal from undiagnosed patients with HCM. This does provide a limiting assumption given the lack of ground truth for disease manifestation but is justified by our sensitivity analysis of this lag time (Figure S9) and previous reports.^27^

## Conclusions

We leveraged population-scale biobank and clinical cohort data to identify novel (ANGPT2 and LTBP2) and known (eg, NT-proBNP and troponins) plasma protein biomarkers with diverse functional relevance in HCM. We further demonstrated their association with incident diagnoses, cardiac imaging markers of disease severity, and adverse clinical outcomes. This highlights the utility of plasma protein profiling in aiding risk and severity prediction in HCM and paves the way for further studies and mechanistic interrogation to clarify the link between HCM and these novel biomarkers.

## Article Information

### Acknowledgments

Authors acknowledge the computational resources provided by the Biomedical Research Computing Facility at the University of Oxford and guidance from Professor Qiang Zhang. Drs Neubauer and Watkins acknowledge support from the Oxford NIHR Biomedical Research center. HCMR (Hypertrophic Cardiomyopathy Registry) was supported by the National Institutes of Health (NIH), the National Heart, Lung, and Blood Institute (U01HL117006-01A1), Cytokinetics, and the Thomas Fund. This research has been conducted using the UK Biobank Resource under Application Number 11223. The authors acknowledge All of Us participants for their contributions and thank the NIH’s All of Us Research Program for making available the examined data. We also acknowledge deCODE Genetics and thank them for the use of their SomaScan V4 study data.

### Disclosures

None.

### Supplemental Material

Supplemental Methods

Tables S1–S7

Figures S1–S10

Supplemental.xlsx File (ST8)

References 28–36

## Supplementary Material

## Appendix

### HCMR Investigators

#### Executive Committee:

Christopher M. Kramer MD, Stefan Neubauer MD, William Weintraub MD, Hugh Watkins MD, PhD, Carolyn Ho MD, Raymond Kwong MD, Milind Desai MD, John DiMarco MD, PhD, Paul Kolm PhD

#### NHLBI:

Patrice Desvigne-Nickens MD, Nancy Geller PhD, Dong-Yun Kim PhD

#### Events Committee:

John DiMarco MD (Chair), Mark Link MD, Gary Francis MD, Barry Greenberg MD

#### HCMR Site Investigators:

Cleveland Clinic Milind Desai MD

Oxford University UK Masliza Mahmod MD DPhil, Betty Raman MD DPhil

Yale Daniel Jacoby MD, Lauren Baldassarre MD

Berlin – Charite, Germany Jeanette Schulz-Menger MD

Calgary, Canada James White MD

London - Kings College, UK Amedeo Chiribiri MD, PhD

University of Michigan Adam Helms MD

Northwestern University Lubna Choudhury MD

Erasmus, the Netherlands Michelle Michels MD

Birmingham, UK William Bradlow MD

UVA Health System Michael Salerno MD, PhD, Christopher Kramer MD

OHSU Steven Heitner MD, Ahmad Masri MD

London – Royal Brompton, UK Sanjay Prasad MD

London - Chest Hospital, UK Saidi Mohiddin MD

Leeds, UK Sven Plein MD

Tufts Medical Center Martin Maron MD, Christopher Madias MD

Stuttgart, Germany Heiko Mahrholdt MD

Bristol, UK Chiara Bucciarelli-Ducci MD, Angus Nightingale MD

Cornell Jonathan Weinsaft MD

Duke Han Kim MD

Leicester, UK Gerry P. McCann MD

Amsterdam, the Netherlands Albert van Rossum MD, Tjeerd Germans MD

Mayo Clinic Eric Williamson MD, Jeff Geske MD

Southampton, UK Andrew Flett MD

Aberdeen, UK Dana Dawson MD

Montréal Heart Institute, Canada F. Pierre Mongeon MD

Florence, Italy Iacopo Olivotto MD

University of Toronto, Canada Andrew Crean MD, Anna Woo MD

University of Pennsylvania Anjali Owens MD

Brigham Carolyn Ho MD

London - St. Georges, UK Lisa Anderson MD, Sanjay Sharma MD

Bologna, Italy Elena Biagini MD

Edinburgh, UK David Newby MD

Heidelberg, Germany Florian Andre MD

Glasgow, UK Colin Berry MD

St. Luke’s Mt. Sinai Bette Kim MD

Quebec City, Canada Eric Larose MD

Johns Hopkins University Theodore Abraham MD, Allison Hays MD

New York University Mark Sherrid MD

Beth Israel Deaconess Evan Appelbaum MD, Eli Gelfand MD

Methodist Hospital, Houston Sherif Nagueh MD

Milan, Italy Ornella Rimoldi MD

McGill University, Canada Eleanor Elstein MD, Matthias Friedrich MD

Rome Sapienza, Italy Camillo Autore MD
