# Supplementary material for: Biobank-Scale Plasma Proteomics Identifies Novel Biomarkers in Hypertrophic Cardiomyopathy
Source: Circ Genom Precis Med. 2026 Apr 22;19(3):e005325. doi: 10.1161/CIRCGEN.125.005325 (PMC13263041; doi:10.1161/CIRCGEN.125.005325)
Supplement: Supplementary file 2 [file hcg-19-e005325-s002.pdf]

SUPPLEMENTAL MATERIAL

Supplemental Methods

Datasets

The National Heart, Lung, and Blood Institute Hypertrophic Cardiomyopathy Registry (HCMR) is a prospective cohort study of 2755 patients with clinically diagnosed HCM enrolled across 44 sites in 6 countries in North America and Europe between April 2014 and April 2017. These patients were phenotyped across a range of modalities including clinical metrics, cardiac magnetic resonance (CMR) imaging, serum biomarkers and genetics[6]. Patients were genotyped by genome-wide genotyping array[28] and amplicon-based sequencing was also performed for 36 cardiomyopathy-associated genes to evaluate rare disease-associated variants[6]. Inclusion and exclusion criteria have been detailed previously[6]. After filtering out individuals who had withdrawn since enrolment, had missing data, were closely related (<3 degrees of relatedness), and did not pass genotyping or sequencing quality control (QC), there remained 2476 HCM cases.

The UK Biobank (UKB) is a longitudinal cohort of ~0.5 million individuals of primarily European ancestry from the United Kingdom between the ages of 40 and 69 at recruitment from 2006 to 2010. Their phenotypic and genotypic data were collected via questionnaire, physical measurements, electronic health records, whole-genome sequencing and genotyping[9]. Approximately 50,000 of these individuals also underwent plasma proteomic analysis using their blood samples collected at recruitment[9]. After filtering for non-NA values in covariates such as age, sex, body mass index (BMI) and more (full list below in Statistical Analyses), there remain 49,588 individuals. From these individuals, HCM cases were identified by self-report from verbal interview, hospital diagnoses (ICD10 and ICD9 codes I42.1/2 and 425.1 respectively) and death cause records (N=100). Heart failure (HF) patients were also identified from the same sources (ICD10 codes I50.0/1/9 and ICD9 codes 4280/4281/4289) (N=2615).

The deCODE Genetics SomaScan v4 cohort is a cohort of ~40,000 Icelandic individuals with health care records and plasma proteins measured as part of the Icelandic Cancer Project and deCODE Genetics programs from 24 August 2000 to 11 January 2019[26,29]. After filtering for non-NA values in covariates, there remain 38,955 individuals. From these individuals, HCM cases were identified by clinical diagnoses (ICD10 and ICD9 codes I42.1/2 and 425.1 respectively) and likewise with HF cases (ICD10 codes I50.0/1/9 and ICD9 codes 4280/4281/4289).

All of Us (AoU) v8 is a longitudinal cohort of >0.6 million individuals of diverse ancestries and backgrounds across the USA enrolled from 2018 to October 2023. Phenotypic data

for these individuals is available in the form of electronic health records (EHR), physical measurements and survey data[30]. From the 393,596 individuals with EHR data available, HCM and HF cases were identified by EHR-derived ICD10 I42.1/2 codes and I50 respectively (N=1482 and 26629 respectively).

Given that plasma proteomic measurements correspond to the individual's state at blood sample collection, cases were divided into 'prevalent' cases (cases diagnosed at or prior to collection date) and 'incident' cases (cases diagnosed after collection date). For HCM however, it is known that only a fraction of cases are diagnosed[1], likely due to their asymptomatic nature, and delays in diagnoses are not uncommon due to misdiagnoses[27]. As such, we assume a lag time in diagnosis after blood sample collection and divide total cases into 'prevalent' and 'incident' cases at this lag time after blood collection. Given the unavailability of a ground truth (true date of HCM manifestation), we assume a lag time of 5 years and demonstrate that results are maintained in sensitivity analyses across a range of possible lag times (Figure S9). For analyses in the deCODE cohort, 'prevalent' cases were classified as those diagnosed at collection date due to the unavailability of diagnosis dates.

Plasma protein assays

The plasma protein assay protocols for HCMR[6], UKB[9] and deCODE[29] have been previously detailed. In brief, HCMR patient-derived blood samples were collected by phlebotomy, held on ice and processed to serum and EDTA-anticoagulated plasma within 1 hour. Samples were assayed by the Biomarker Research and Clinical Trials Laboratory at Brigham & Women's Hospital for the following biomarkers: N-terminal prohormone of brain natriuretic peptide (NTproBNP) via Roche proBNP II assay (5-35,000 pg/mL analytical range) and cardiac troponin T (TnT) via Roche TnT STAT Gen 5 assay (6 – 10,000 ng/L analytical range).

For UKB[9], blood samples were processed to EDTA plasma, buffy coat and red blood cells, and shipped on dry ice to Olink Analysis Service in Sweden. The Olink Explore 3072 panel was used with quality control (QC) as per internal Olink procedures[9]. Output data was generated in Normalised Protein eXpression (NPX) values, reflecting a unit of relative quantification (to a reference control) on a log-2 scale. Following such QC[9], 2922 plasma proteins were evaluated.

For deCODE[29], blood samples were processed to plasma and measured using the SomaScan v4 assay (5284 aptamers). QC was performed as described previously[29] resulting in 4963 aptamers evaluated in this plasma proteomic study. Each aptamer specifically binds a target protein with high affinity, such that measuring the abundance of bound aptamers via DNA microarrays provides a proxy for their target protein's abundance in the plasma. This provides output as relative fluorescence units (to a reference control).

AoU provides EHR-linked plasma protein measurements including NTproBNP, brain natriuretic peptide (NPPB), TnT and Troponin I (TnI). Given their EHR basis, measurements were not collected in a standardised fashion across all individuals. Due to heterogeneity arising from varying assay kits, lab processing and other potential batch effects, quality control was performed via filtering out of non-concentration-based (e.g. activity) measurements, standardisation of concentration measurements to ng/mL, filtering out outliers >2000 ng/mL, and subsequently removing outliers >3 standard deviations from the mean. This 2000 ng/mL upper threshold was chosen as it greatly exceeds the upper limit of detection for representative assays (NTproBNP: Roche proBNP II assay = 35 ng/mL; NPPB: Abbott ARCHITECT BNP assay = 5 ng/mL; TnT: Roche TnT STAT Gen 5 assay = 10 ng/mL; TnI: Abbott ARCHITECT STAT High-Sensitivity Troponin I assay = 5ng/mL) even after considering potential dilution of samples. Subsequently, the 3 standard deviation upper thresholds applied for NTproBNP, NPPB, TnT and TnI are respectively 37.1, 23.1, 14.6, 30.3 ng/mL. For multiple measurements in a single individual, the most recent measurement as per the corresponding EHR record was taken. Following such QC, 41595 individuals had at least one non-NA plasma protein measurement of interest, of which 546 were HCM cases.

Cardiac magnetic resonance imaging phenotypes

Association analysis between plasma proteins and CMR imaging-derived endophenotypes of disease severity was performed to evaluate cross-phenotype associations. The CMR protocol for HCMR has been previously detailed[6] and of the numerous phenotypes measured, a select few were chosen prior to analysis to minimize multiple testing burden. These included maximal LV wall thickness (maxLWVT) and indexed LV mass (LVMi) to reflect hypertrophy; late gadolinium enhancement (LGE) and extracellular volume fraction (ECVF) to measure replacement and interstitial fibrosis respectively; LV ejection fraction (LVEF) and median global transmural longitudinal, circumferential, and radial LV strains to reflect LV contractile function.

Statistical analysis

Exploratory data analysis of plasma proteins and CMR measures was carried out in all datasets. This included distribution and quantile-quantile (QQ) plotting to evaluate normality. Traits with skewed distributions, including all plasma protein measurements, were rank-based inverse normalised prior to downstream analyses. Distributions were also evaluated to filter out null outliers (erroneous values of 0) and physiologically impossible outliers (negative values in non-negative phenotypes such as plasma proteins and CMR measures). Pairwise Spearman rank correlation was evaluated between quantitative independent variables in multivariable association analyses to identify collinearity ($|r| > 0.8$) and if so, one of the pair (the one with more missing

values) was excluded from the analysis. Statistical analyses were conducted in R (v4.3.3).

Case-control differential protein expression analysis was performed across plasma proteins in the UKB, deCODE and AoU via multivariable linear regression with empirical Bayes smoothing via *limma* R package (v3.58.1). In the latter 2 datasets, aforementioned regression without said smoothing was applied due to few proteins tested.

Specifically, models fitted were of the form (weight coefficients absent for ease of interpretation) to evaluate whether the disease status alters plasma protein levels:

$$plasma\ protein\ x \sim HCM\ disease\ status\ +\ covariates + \ \varepsilon$$

HCM disease status was determined by inclusion in 'prevalent' HCM cases, and controls were defined as all non-'prevalent' HCM and non-HF individuals to maximise statistical power. Covariates included age, sex, body mass index (BMI), 5 common genetic principal components (representing genetic ancestry), diastolic blood pressure, type 2 diabetes status, smoking status, social deprivation index, estimated glomerular filtration rate (from creatinine levels as per CKD-EPI[31]), low-density lipoprotein levels, and (in AoU only due to excessive missingness in UKB: 4171/49588 NA values and unavailability in DeCODE Genetics) high-density lipoprotein levels. This comprehensive covariate set was selected to control for environmental, lifestyle, and comorbidity-related factors that act as confounders for circulating protein levels and so, isolate disease-specific associations. Multiple testing correction was applied to moderated t-test p-values via the Benjamini-Hochberg (BH) procedure to control the Type I error rate at 5%. Sensitivity analyses were also performed with non-HF HCM cases and non-HCM HF cases compared to the same controls.

Time-to-event (TTE) analysis was also performed in UKB to identify associations between plasma proteins and a) incident HCM diagnosis (as per UKB case criteria mentioned previously) and b) adverse clinical outcomes. In the former, controls defined above in the case-control analysis (including 'incident' HCM cases) were analysed, whereas in the latter, total cases were analysed to maximise statistical power. In both analyses, multivariable Cox regression was performed with models of the form (weight coefficients absent for ease of interpretation) to evaluate the relationship between baseline plasma protein levels and incident clinical outcomes:

$$H(t) \sim H_0(t) \times e^{plasma\ protein\ x + covariates} + \varepsilon$$

with covariates as per case-control analyses. The timescale used was age to reduce age-related confounding bias in cohort studies[32] with baseline at initial centre attendance. Individuals were left-truncated at this date if they had prior incidence of composite and right-censored at either date of 'lost to follow-up', death or dataset version date (2024-02-09). The proportional hazards assumption was assessed via

visual evaluation of scaled Schoenfeld residuals vs. transformed timescale plot and per-covariate score tests. Linearity was assessed via visual evaluation of Martingale residuals vs. continuous predictors. Kaplan-Meier analyses were also performed for univariate analyses. For select plasma proteins, the top 25% quantile were compared to the bottom 75% by log-rank test to evaluate for significant differences between patients in their respective subgroups. TTE analysis was performed via s*urvival* R package (v.3.5.8). TTE analyses were not performed in the deCODE Genetics dataset due to unavailability of diagnosis and event dates, nor in the AoU dataset due to insufficient incident diagnoses since recruitment.

For differential protein expression and TTE analyses for incident diagnosis in UKB and (for only the former) deCODE, inflation in test statistics was evaluated by quantile-quantile plotting and calculation of inflation factor λ (Table S10). If deviance from the expected uniform distribution of p-values was observed and $\lambda > 1.1$, then chi-squared test statistics were inflation-corrected using division of chi-squared test statistic by λ and p-values recomputed.

Composite clinical outcomes were constructed from clinical events in the UKB's hospital-linked or death cause record-linked ICD10 codes and operations records to organise related outcomes and increase statistical power. The ventricular arrhythmia (VA) composite was defined as first occurrence of resuscitated cardiac arrest (I46), sudden cardiac death (I46.1 in death cause record) and implantable cardioverter-defibrillator therapy (OPCS4 K59 from operations record or self-reported). The heart failure (HF) composite was defined as first occurrence of heart transplant (OPCS4 K01 & K02 from operations record or self-reported) or incident heart failure (I50). An overall composite was constructed defined as first occurrence of any VA or HF events, as well as all-cause mortality, atrial fibrillation (I48) and stroke (I64).

Functional enrichment analysis was performed to evaluate significant enrichment of plasma proteins with certain biological and molecular pathways. Specifically, pathway databases of Gene Ontology: Biological Process & Molecular Function as well as Kyoto Encyclopaedia of Genes and Genomes were used in conjunction with *ClusterProfiler* R package (v4.10.1). Hypergeometric test was applied for over-representation analysis with multiple testing correction via BH procedure given background set of genes corresponding to the 2922 plasma proteins.

Association analysis between plasma proteins and CMR phenotypes in HCMR was performed via multivariable linear regression to adjust for confounders. Specifically, models were fitted of the form (weight coefficients absent for ease of interpretation) to evaluate for whether protein levels can predict clinical phenotype:

$$CMR\ phenotype \sim plasma\ protein\ x\ +\ covariates +\ \varepsilon$$

where the covariates included age, sex, BMI, 5 common genetic principal components, diastolic blood pressure, sarcomeric rare variant carrier status (as previously defined[6]) and for non-indexed, non-percentage-based phenotypes as maxLVWT, body surface area as well. CMR phenotypes were normalised by rank-based inverse normalisation to enable comparison across different phenotypes. Multiple testing correction was applied via BH procedure.

Genome-wide association studies

HCMR patients were genotyped using the Axiom Precision Medicine Research Array (Affymetrix) as previously described[28]. Hard-called variants were aligned to the Haplotype Reference Consortium (HRC) reference panel using HRC-1000G-check-bim-v4.3.0.pl (https://www.chg.ox.ac.uk/~wrayner/tools/). QC filtering for variants was subsequently carried out (MAF > 1%, MAC > 100, genotype missing rate < 1%, Hardy-Weinberg equilibrium test $P > 10^{-15}$) and individuals with > 10% genotype missing rate were also removed. Hard-called variants were subsequently LD pruned (1000 variant windows, 100 sliding windows, $r^2 < 0.8$) using --indep-pairwise function in PLINK (v1.90b6). The Michigan Imputation Server was used to perform haplotype phasing with Eagle and subsequent imputation against HRC (version r1.1.2016) reference panel[28]. 10 common genetic principal components were derived using FlashPCA2 with default settings.

Genotyping of UKB individuals was performed using the UK Biobank Axiom Array with QC, principal component analysis and imputation as previously described[33]. We also excluded 1235 UKB individuals who had been used as controls in the HCM GWAS[34] which we later use to derive HCM instruments for Mendelian randomisation (MR), resulting in 48353 individuals for plasma protein GWAS in UKB. This was necessary to maintain a lack of sample overlap between the exposure and outcome GWASs assumed in two-sample MR. The same QC filters and LD pruning as for HCMR were applied in UKB.

REGENIE (v3.4.1) was used for GWAS via a two-step procedure to account for population structure[35]. In short, step 1 fits a whole-genome ridge regression model for each trait using a leave-one-chromosome-out cross-validation scheme. QC-filtered and LD-pruned hard-called variants were used in this first step. Step 2 uses the 23 LOCO phenotypic predictions derived from step 1 as offsets in linear regression for GWAS against the imputed variants. Only imputed variants with MAF > 1%, MAC > 50 and INFO score > 0.7 were used for Step 2 to reduce spurious associations. Rank-based inverse-normal transformation was also applied to all phenotypes for normalisation. Covariates of age, sex, height, weight and 10 common genetic principal components were used to adjust for population stratification and basic demographic effects. Downstream clinical mediators (e.g., lipids, comorbidities) were excluded from the GWAS covariate set to

avoid over-adjustment and ensure the capture of total genetic effects, including those mediated through physiological pathways.

Visualisation of GWAS results was performed via Manhattan plotting (*ggmanh* R package) and LocusZoom plotting of regions with at least 2 variants with p-value $<10^{-5}$ (for HCMR) or 5×10^{-8} (for UKB) in a span of 10^6 base pairs. QC of results was also performed via evaluation of p-value histogram, quantile-quantile plot (against chi-squared distribution) and genomic inflation factor (λ). Genomic regions passing the LocusZoom criteria as above were fine-mapped to identify causal variant(s) within each region via FINEMAP (v1.4.2) with default settings. LDSTORE2 (v2.0) was used to compute in-sample LD statistics for each genomic region of interest. Top-ranked causal configurations for each genomic region with a $\log_{10}$(Bayes Factor) > 0 were isolated and their variants passed to Open Target Genetics API for variant-to-gene (V2G) prioritisation via *otargen* R package (v1.1.1). If variants were not in rsID format, genomic coordinates were lifted over from Hg19 to Hg38 using *liftOver* R package (v1.2.2). Prioritised genes were evaluated and manually curated to identify *cis*-pQTLs (protein quantitative trait loci) defined as variants residing within 1Mbp up or downstream of the transcription start site of the gene encoding the protein.

Mendelian randomisation

Mendelian randomisation was performed to evaluate the causality of exposure on outcome variable using *TwoSampleMR*[36] (v0.6.11) R package. A two-sample approach was utilised to improve statistical power by leveraging multiple datasets and mitigate weak instrument bias[12]. A bidirectional approach was also used to clarify the direction of potential causal relationships.

Instrument selection procedure varied for the exposure variable. For plasma proteins, genetic instruments from respective GWASs were selected from the top-ranked causal configuration for each genomic region passing aforementioned LocusZoom criteria. Instruments from these top-ranked configurations must individually have p-value $<10^{-5}$ (for HCMR) or 5×10^{-8} (for UKB). Additionally, only *cis*-pQTLs were selected as such variants are most likely to satisfy instrumental variable assumptions because they directly encode the plasma protein or its precursor(s)[12]. If such *cis*-pQTLs are unavailable, *trans*-pQTLs linked to functionally relevant genes by V2G prioritisation were used instead.

For hypertrophic cardiomyopathy disease status as the exposure variable, 68 HCM-associated lead variants from a recent multi-trait-analysis (MTAG) GWAS meta-analysis[34] were selected. For MR, a 2-sample approach was utilised which assumes lack of sample overlap in exposure and outcome GWASs. If the outcome GWAS utilised UKB individuals, the 68 variant rsIDs were derived from the MTAG GWAS but the beta and standard errors for these instruments were derived from the non-MTAG GWAS to

prevent sample overlap due to the use of UKB-derived GWASs in MTAG[34]. This approach reduced overfitting from winner's curse (by utilising non-MTAG summary statistics for MR) while still leveraging the increased statistical power of the MTAG approach for variant discovery. To ensure validity of the relevance assumption for instrumental variables, an additional p-value filter of 10^{-5} in the non-MTAG GWAS was applied, reducing the number of HCM instruments to 55[12]. If instead the outcome GWAS utilised HCMR individuals, HCM instruments were restricted to 19 variants passing genome-wide-significance (GWS) ($p < 5 \times 10^{-8}$) in the HCMR-less GWAS of HCM[34] to prevent sample overlap. Variants selected as instruments were evaluated for excessive pleiotropy by PheWAS in UK Biobank, FinnGen Consortium and GWAS Catalog via Open Target Genetics (v22.10) and removed if so.

Mendelian randomisation was carried out with inverse-variance-weighted (IVW) method (if multiple instruments) or via Wald ratio estimate (if single instrument) as per recent guidelines[12]. For binary exposures, causal estimates were also transformed to reflect increase in outcome per doubling of genetic liability of exposure (assuming binary exposure is rare so odds $\approx$ probability). Sensitivity analyses to assess heterogeneity were conducted using single-instrument and leave-one-out analyses as well as statistical test of Cochran's Q, scatter and funnel plotting. Robust MR methods for sensitivity analysis were conducted using MR Egger regression, weighted median and weighted mode. Directional horizontal pleiotropy was also evaluated via statistical test of MR Egger regression intercept inequality from 0.

Colocalisation analyses were also performed for sensitivity analysis to evaluate if both exposure and outcome traits had shared associated causal variant(s) at each locus via *coloc* R package (v5.2.3). Specifically, HCM genetic instruments' loci (500kbp window on each side of lead variant) were analysed using the UKB EUR ancestry LD matrix between HCM and outcome phenotype GWASs.

Machine learning for joint modelling

Case-control and TTE statistical analyses applied marginal models where each plasma protein was individually modelled as opposed to jointly modelled together. Machine learning (ML) models enable joint modelling of plasma proteins to capture potential non-linear relationships and interaction effects. This enables inference of relative importance of plasma proteins to prediction of case status in UKB. Given this particular aim of inference (as opposed to prediction), the entire dataset of prevalent cases and controls was used for training.

Input features included clinical covariates (as per case-control analysis) and plasma proteins passing the 5% FDR significance threshold from said analysis. This feature selection to the subset of plasma proteins (as opposed to all 2922) was used to 1) prevent overfitting on noise from non-disease-linked biomarkers, 2) focus on the joint

inference of these biologically relevant candidate biomarkers and 3) provide greater efficiency from a potential translational perspective.

Feature preprocessing included imputation of missing plasma protein values via K-nearest-neighbours approach (K=5) and one-hot-encoding of categorical values. These steps were applied within a stratified 5-fold cross-validation (CV) scheme. Extreme gradient-boosted tree-based ensemble modelling (XGBoost) was used for binary classification due to its robustness to varying scales and data types for features, and robustness to case-control imbalance. Training was performed within the CV scheme to strictly regularise model complexity to ensure the validity of inference and mitigate risk of overfitting. Hyperparameters controlling model complexity (n_estimators, learning_rate and scale_pos_weight) were optimised by grid search within the CV scheme to maximise area under the receiver operating curve (AUC) scoring metric.

The best-performing model by average validation-fold AUC (highest generalisability) was retrained on the entire training set with relative feature importance estimated by Shapley additive explanation (SHAP) values[14]. Specifically, per-feature mean |SHAP| values were computed with 95% confidence intervals estimated by bootstrapping the background dataset (iterations = 1000) to derive a distribution of mean |SHAP| values per feature. For unbiased interpretation in the presence of case-control imbalance, SHAP values were computed for cases and controls separately. SHAP values were further decomposed to SHAP interaction values to evaluate pairwise interaction effects between plasma proteins. ML analyses were performed in Python (v3.11.6) using *scikit-learn* (v1.3.2), *shap* (0.46.0) and *xgboost* (2.1.3) packages.

Supplemental Tables

	Cases		Controls	
**Phenotype**	Summary Statistic	Non-NA Counts	Summary Statistic	Non-NA Counts
Age	61.00 [58.00 - 65.00] years	42	58.00 [50.00 - 64.00] years	49096
Sex		42		49096
Male	25 (59.5%)		22479 (45.8%)	
Female	17 (40.5%)		26617 (54.2%)	
Self-Reported Ethnicity		42		49096
Asian	<5		1039 (2.1%)	
Black	<5		1103 (2.2%)	
Mixed	<5		330 (0.7%)	
Other	<5		563 (1.1%)	
Unknown/Not Stated	<5		170 (0.3%)	
White	40 (95.2%)		45891 (93.5%)	
Body Mass Index	28.72 [25.94 - 30.83] kg/m^2	42	26.75 [24.16 - 29.89] kg/m^2	49096
Diastolic Blood Pressure	83.01 ± 12.27 mmHg	42	83.36 ± 10.94 mmHg	49096
Type 2 Diabetes	<5	42	1401 (2.9%)	49096
Smoking		42		49096
Current	<5		5208 (10.6%)	
Previous	16 (38.1%)		17119 (34.9%)	
Never	22 (52.4%)		26769 (54.5%)	
Townsend Deprivation Index	-2.00 [-3.48 - 0.44]	42	-2.07 [-3.63 - 0.72]	49096
Estimated Glomerular Filtration Rate	5.12 [3.29 - 5.29] $mL/min/1.73\ m^2$	42	3.64 [3.32 - 5.29] $mL/min/1.73\ m^2$	49096
Low-Density Lipoprotein	3.36 ± 0.95 mmol/L	42	3.54 ± 0.88 mmol/L	49096
**Plasma Proteins**				
NTproBNP	2.66 ± 2.22 SD	42	0.07 ± 1.22 SD	47696
BNP	2.07 ± 2.72 SD	41	-0.05 ± 1.51 SD	46841
Troponin I	0.65 ± 1.00 SD	41	-0.08 ± 0.94 SD	46841
LTBP2	0.33 ± 0.45 SD	42	0.00 ± 0.42 SD	48290
ANGPT2	-0.22 ± 0.41 SD	42	-0.01 ± 0.35 SD	47824

Table S1. Summary of demographic, covariate and plasma proteins for cases and controls from case-control analysis in UK Biobank. *Cases here defined as 'prevalent' HCM cases given 5 year lag time assumption and controls defined as non-prevalent 'HCM' and non-HF individuals. Normally distributed traits are summarised by mean ± standard deviation whereas non-normally distributed traits are summarised with median [IQR]. Rank-based inverse normalised traits (such as plasma proteins) have units of SD. SD: standard deviation.*

	Cases		Controls	
**Phenotype**	Summary Statistic	Non-NA Counts	Summary Statistic	Non-NA Counts
Age	70.30 [61.30 - 77.30] years	546	65.30 [55.30 - 74.30] years	31336
Sex		546		31336
Male	301 (55.1%)		12841 (41.0%)	
Female	245 (44.9%)		18495 (59.0%)	
Genetically Predicted Ancestry		546		31336
African	140 (25.6%)		6885 (22.0%)	
American	43 (7.9%)		4188 (13.4%)	
East Asian	<20		377 (1.2%)	
European	346 (63.4%)		19582 (62.5%)	
Middle Eastern	<20		98 (0.3%)	
South Asian	<20		206 (0.7%)	
Body Mass Index	29.70 [26.20 - 34.48] kg/m^2	546	29.80 [25.80 - 35.10] kg/m^2	31336
Diastolic Blood Pressure	76.98 ± 13.15 mmHg	546	77.96 ± 11.13 mmHg	31336
Type 2 Diabetes	248 (45.4%)	546	10391 (33.2%)	31336
Townsend Deprivation Index	0.30 [0.26 - 0.33]	546	0.30 [0.28 - 0.34]	31336
Estimated Glomerular Filtration Rate	84.71 [68.86 - 101.18] mL/min/1.73 m^2	546	92.10 [76.74 - 107.28] mL/min/1.73 m^2	31336
Low-Density Lipoprotein	100.00 [75.25 - 127.00] mg/dL	546	107.00 [83.00 - 131.60] mg/dL	31336
High-Density Lipoprotein	46.00 [38.00 - 57.00] mg/dL	546	49.00 [40.00 - 60.00] mg/dL	31336
**Plasma Proteins**				
NTproBNP	0.51 ± 0.93 SD	307	-0.30 ± 0.87 SD	12301
BNP	0.47 ± 0.86 SD	221	-0.34 ± 0.87 SD	6173
Troponin T	0.39 ± 0.98 SD	310	-0.09 ± 0.95 SD	17366
Troponin I	0.38 ± 0.94 SD	246	-0.17 ± 0.92 SD	10742

Table S2. Summary of demographic, covariate and plasma proteins for cases and controls from case-control analysis in All of Us (v8). *Cases here defined as 'prevalent' HCM cases given 5 year lag time assumption and controls defined as non-prevalent 'HCM' and non-HF individuals. Normally distributed traits are summarised by mean ± standard deviation whereas non-normally distributed traits are summarised with median [IQR]. Rank-based inverse normalised traits (such as plasma proteins) have units of SD. SD: standard deviation.*

Phenotype	Summary Statistic	Non-NA Counts
Age	52.00 [43.00 - 58.00] years	2476
Sex		2476
Male	1786 (72.1%)	
Female	690 (27.9%)	
Self-Reported Ethnicity		2474
American Indian/Alaskan	10 (0.4%)	
Black/African American	183 (7.4%	
East Asian	60 (2.4%)	
Pacific Islander	8 (0.3%)	
South Asian	134 (5.4%)	
White	2079 (84.0%)	
Body Mass Index	28.40 [25.35 - 32.42] kg/m^2	2476
Body Surface Area	2.01 [1.86 - 2.17] m^2	2359
Diastolic Blood Pressure	84.00 [75.00 - 93.00] mmHg	2179
Sarcomeric Variant Carrier Status	848 (34.7%)	2441
**CMR Imaging-Derived LV Parameters**		
Maximal LV Wall Thickness	20.02 [17.22 - 23.27] mm	2359
Indexed LV Mass	80.82 [66.64 - 99.07] g/m^2	2359
LV Ejection Fraction	64.64 [58.78 - 70.03] %	2359
Global Average Longitudinal Strain	11.00 [9.14 - 12.65]	2104
Global Average Circumferential Strain	11.61 [10.00 - 13.28]	2104
Global Average Radial Strain	33.27 [23.53 - 44.90]	2104
Cardiac Index	3.23 [2.83 - 3.69] L/min/m^2	2359
Total LGE Over All Segments	0.08 [0.00 - 1.61] %	2331
Whole LV ECVF	29.43 [26.48 - 32.72] %	1864
**Plasma Proteins**		
NTproBNP	0.00 ± 1.00 SD	2465
Troponin T	0.01 ± 0.96 SD	2465

Table S3. Summary of demographic, covariate, cardiac magnetic resonance (CMR) imaging measures and plasma proteins in cases from Hypertrophic Cardiomyopathy Registry (HCMR). *Normally distributed traits are summarised by mean ± standard deviation whereas non-normally distributed traits are summarised with median [IQR]. Rank-based inverse normalised traits (such as plasma proteins) have units of SD. ECVF: Extracellular volume fraction, LV: Left ventricle, SD: standard deviation.*

Composite Outcome	Outcome Component	Number Of First Events
VA	ICD Implantation	11
	Resuscitated Cardiac Arrest (i46)	<5
	SCD (i46.1)	<5
HF	HF (i50)	33
	Heart Transplantation	<5
Overall	Atrial Fibrillation (i48)	30
	HF (i50)	16
	ICD Implantation	<5
	Resuscitated Cardiac Arrest (i46)	<5
	Death	<5
	Stroke (i64)	<5
	SCD (i46.1)	<5
	Heart Transplantation	<5

Table S4. Number of adverse clinical event outcomes for each composite across 100 HCM cases in UK Biobank. *Individuals were left-truncated at time of initial centre attendance if they had prior incidence of composite outcome. Median follow-up times (years) for each of the composites were 11.2 [IQR, 7.5 – 14.5] for overall, 14.3 [IQR, 9.7 – 15.4] for HF and 14.7 [13.9 – 15.5] for ventricular arrhythmia (VA).*

Exposure	Outcome	Number of SNPs	Beta	Standard Error	p-value	Plasma Protein GWAS Dataset
HCM	ANGPT2	3	-0.151	0.124	0.225	UKB
ANGPT2	HCM	55	0.009	0.008	0.248	UKB
LTBP2	HCM	55	-0.004	0.006	0.571	UKB
**NPPB**	**HCM**	**55**	**0.030**	**0.013**	**0.015**	**UKB**
**NTproBNP**	**HCM**	**55**	**0.033**	**0.016**	**0.044**	**UKB**
TNNI3	HCM	55	0.009	0.007	0.176	UKB
HCM	LTBP2	3	-0.112	0.174	0.521	UKB
HCM	NPPB	2	0.140	0.106	0.188	UKB
HCM	NTproBNP	3	0.091	0.076	0.229	UKB
**NTproBNP**	**HCM**	**19**	**0.147**	**0.040**	**2.11E-04**	**HCMR**
**TNNT2**	**HCM**	**19**	**0.095**	**0.033**	**0.004**	**HCMR**
HCM	NTproBNP	1	0.034	0.185	0.854	HCMR
HCM	TNNT2	2	0.105	0.201	0.602	HCMR

Table S5. Bidirectional, 2-sample Mendelian randomisation analyses results across plasma proteins and HCM disease status. *p-values are reported above prior to multiple testing correction. Bold entries indicate nominally significant relationships. * indicates Wald ratio method used instead of inverse-variance-weighted method.*

GWAS Dataset	Exposure	rsID	Effect Allele	Non Effect Allele	Effect Allele Frequency	Beta (Exposure)	Standard Error (Exposure)	p-value	Sample Size
UKB	ANGPT2	rs1968586	T	C	0.340	-0.146	0.007	1.67E-105	47615
	ANGPT2	rs2959812	G	A	0.715	-0.128	0.007	3.23E-77	47615
	ANGPT2	rs9314610	C	T	0.445	-0.042	0.006	1.59E-11	47615
	LTBP2	rs11159087	T	C	0.155	-0.096	0.007	1.16E-38	48104
	LTBP2	rs4899522	A	G	0.353	-0.053	0.006	4.19E-21	48104
	LTBP2	rs888414	A	G	0.383	-0.104	0.005	6.36E-83	48104
	NPPB	rs198389	G	A	0.420	0.181	0.006	3.87E-173	48128
	NPPB	rs2076003	C	T	0.051	0.218	0.014	3.70E-52	48128
	NTproBNP	rs198379	C	T	0.414	0.254	0.006	1.00E-200	48128
	NTproBNP	rs41300100	G	C	0.010	0.573	0.033	6.30E-68	48128
	NTproBNP	rs79593079	T	C	0.055	0.271	0.013	5.58E-96	48128
HCM Meta-analysis (non-MTAG)	HCM	rs1009358	T	C	0.614	0.111	0.022	3.88E-07	74259
	HCM	rs1048302	T	G	0.326	0.253	0.022	8.47E-30	74259
	HCM	rs10841524	A	G	0.624	-0.118	0.023	2.91E-07	74259
	HCM	rs11196085	T	C	0.723	-0.203	0.024	1.85E-17	74259
	HCM	rs113907726	T	G	0.813	-0.152	0.028	4.10E-08	74259
	HCM	rs11687178	C	A	0.651	0.135	0.023	7.70E-09	74259
	HCM	rs11748963	T	C	0.714	-0.157	0.024	7.26E-11	74259
	HCM	rs11860560	G	A	0.927	-0.206	0.042	1.13E-06	74259
	HCM	rs12210733	G	A	0.945	-0.418	0.043	5.42E-22	74259
	HCM	rs12270374	T	C	0.639	-0.140	0.023	6.85E-10	74259
	HCM	rs12460541	G	A	0.662	0.150	0.023	6.01E-11	74259
	HCM	rs13297385	C	T	0.722	-0.124	0.024	1.83E-07	74259
	HCM	rs139920	C	T	0.486	-0.113	0.023	1.09E-06	74259
	HCM	rs140763915	C	T	0.983	-0.380	0.079	1.34E-06	74259
	HCM	rs17617337	C	T	0.790	-0.373	0.025	1.87E-49	74259
	HCM	rs17817857	C	T	0.736	-0.129	0.025	3.64E-07	74259
	HCM	rs182427065	G	A	0.987	-1.231	0.096	2.48E-37	63580
	HCM	rs2002854	A	G	0.262	0.129	0.024	1.22E-07	74259
	HCM	rs2177843	C	T	0.841	-0.235	0.030	2.80E-15	74259
	HCM	rs2182400	A	G	0.761	0.115	0.026	8.74E-06	74259
	HCM	rs2184370	G	A	0.643	-0.105	0.022	3.07E-06	74259
	HCM	rs2191446	G	A	0.205	-0.214	0.029	8.25E-14	74259
	HCM	rs2503715	A	G	0.129	-0.211	0.043	1.12E-06	69476
	HCM	rs2540277	C	T	0.937	0.278	0.050	2.31E-08	74259
	HCM	rs2644262	T	C	0.712	-0.329	0.024	1.79E-43	74259
	HCM	rs2645210	A	G	0.189	0.153	0.028	3.94E-08	74259

	HCM	rs2810883	C	T	0.461	0.104	0.022	1.89E-06	74259
	HCM	rs28575249	G	A	0.495	0.102	0.022	3.11E-06	74259
	HCM	rs28768976	A	G	0.766	-0.236	0.025	7.43E-21	74259
	HCM	rs2945236	G	A	0.479	-0.101	0.022	3.70E-06	74259
	HCM	rs3176326	G	A	0.788	-0.267	0.026	3.18E-24	72592
	HCM	rs35006907	C	A	0.691	-0.152	0.023	9.63E-11	74259
	HCM	rs3845778	C	T	0.519	-0.153	0.022	1.93E-12	74259
	HCM	rs41306688	A	C	0.967	-0.471	0.060	3.04E-15	74259
	HCM	rs4894803	A	G	0.594	-0.171	0.023	2.19E-13	74259
	HCM	rs547048	T	C	0.203	0.138	0.027	2.49E-07	74259
	HCM	rs5760054	C	T	0.222	0.268	0.026	6.62E-25	74259
	HCM	rs58747679	T	C	0.710	0.118	0.024	1.30E-06	74259
	HCM	rs62222424	G	A	0.934	0.279	0.046	1.21E-09	74259
	HCM	rs6566955	A	G	0.695	-0.132	0.024	2.93E-08	74259
	HCM	rs66520020	C	T	0.838	-0.195	0.030	5.87E-11	74259
	HCM	rs6699769	A	G	0.794	0.125	0.027	4.03E-06	74259
	HCM	rs6747402	G	A	0.524	0.129	0.022	4.14E-09	74259
	HCM	rs6796333	T	C	0.819	0.153	0.029	8.02E-08	74259
	HCM	rs6914805	C	T	0.730	0.132	0.024	5.02E-08	74259
	HCM	rs7210446	G	A	0.426	-0.214	0.023	3.88E-21	74259
	HCM	rs74139614	C	T	0.954	-0.262	0.051	3.33E-07	74259
	HCM	rs7487962	A	G	0.259	-0.204	0.026	1.43E-15	74259
	HCM	rs75096272	G	A	0.965	-0.281	0.054	2.29E-07	74259
	HCM	rs7612736	G	A	0.792	0.199	0.028	1.40E-12	74259
	HCM	rs7824244	G	A	0.862	-0.199	0.031	2.39E-10	74259
	HCM	rs7835298	G	A	0.563	-0.103	0.022	3.28E-06	74259
	HCM	rs8006225	G	T	0.825	0.203	0.030	2.64E-11	74259
	HCM	rs8033459	C	T	0.539	-0.187	0.022	7.04E-18	74259
	HCM	rs816379	A	C	0.628	0.121	0.024	4.60E-07	74259

Table S6. Genetic instrument details used for Mendelian randomisation analyses with UK Biobank-derived plasma protein GWASs.

GWAS Dataset	Exposure	rsID	Effect Allele	Non Effect Allele	Effect Allele Frequency	Beta (Exposure)	Standard Error (Exposure)	p-value	Sample Size
HCMR	NTproBNP	rs198388	T	C	0.460	0.139	0.027	2.94E-07	2465
	TnT	rs11418951	CA	C	0.232	0.137	0.030	6.12E-06	2465
	TnT	rs72798626	T	G	0.016	0.464	0.101	4.47E-06	2465
HCMR-less HCM Meta-analysis (non-MTAG)	HCM	rs1048302	G	T	0.674	-0.243	0.027	6.71E-20	34914
	HCM	rs11196085	C	T	0.275	0.220	0.028	9.09E-15	34914
	HCM	rs11748963	C	T	0.287	0.180	0.028	2.54E-10	34914
	HCM	rs12210733	A	G	0.057	0.390	0.053	1.17E-13	34914
	HCM	rs17617337	T	C	0.212	0.355	0.030	8.97E-32	34914
	HCM	rs182427065	A	G	0.015	1.630	0.130	7.05E-36	20866
	HCM	rs2177843	T	C	0.156	0.240	0.034	1.84E-12	34914
	HCM	rs2191446	A	G	0.788	0.214	0.033	1.06E-10	34914
	HCM	rs2644262	C	T	0.291	0.326	0.028	2.35E-31	34914
	HCM	rs28768976	G	A	0.244	0.222	0.030	1.40E-13	34914
	HCM	rs3176326	A	G	0.216	0.298	0.032	1.65E-20	33247
	HCM	rs3845778	T	C	0.478	0.143	0.026	2.41E-08	34914
	HCM	rs41306688	C	A	0.033	0.413	0.069	2.51E-09	34914
	HCM	rs4894803	G	A	0.408	0.171	0.027	2.05E-10	34914
	HCM	rs5760054	T	C	0.771	-0.240	0.031	1.38E-14	34914
	HCM	rs66520020	T	C	0.160	0.195	0.035	2.14E-08	34914
	HCM	rs7210446	A	G	0.566	0.213	0.026	1.02E-16	34914
	HCM	rs8006225	T	G	0.178	-0.208	0.034	1.12E-09	34914
	HCM	rs8033459	T	C	0.450	0.197	0.026	1.54E-14	34914

Table S7. Genetic instrument details used for Mendelian randomisation analyses with HCMR-derived plasma protein GWASs.

Table S8. Differential protein expression case-control analysis results from UK Biobank for 2922 plasma proteins.

Supplemental Figures with Figure Legends

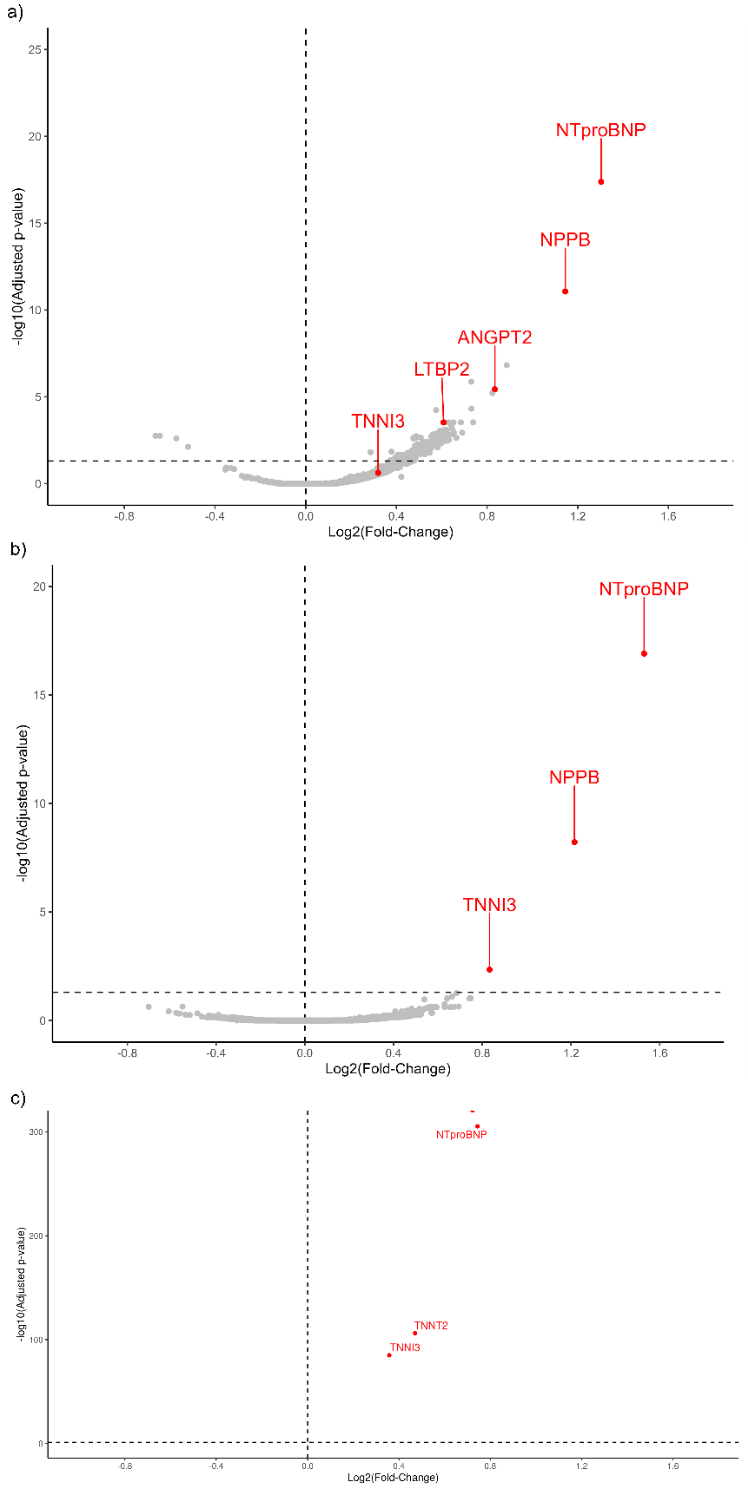

Figure S1.Case-control differential protein expression analyses of non-HCM heart failure cases and non-HF HCM cases in UK Biobank and All of Us datasets. a) *Case-control analysis in 450 UK Biobank heart failure patients excluding HCM cases shows that of the 5 plasma proteins significantly associated with HCM, 4 are also significantly associated with heart failure, reflecting the shared pathobiology underlying these two diseases.* ***b)*** *Case-control analysis in 37 HCM patients excluding heart failure cases shows that 3 of the 5 plasma proteins significantly associated with overall HCM are replicated in the HF-free analysis.* ***c)*** *Case-control analysis of All of Us heart failure patients excluding HCM cases also shows significant association of known biomarkers brain natriuretic peptide (NPPB), NTproBNP, and Troponins I & T with heart failure as well. The obscured NPPB point reflects its adjusted p-value is outside the limits of precision for numerics in R (1.8 x 10^{308}). TNNI3: Troponin I, TNNT2: Troponin T.*

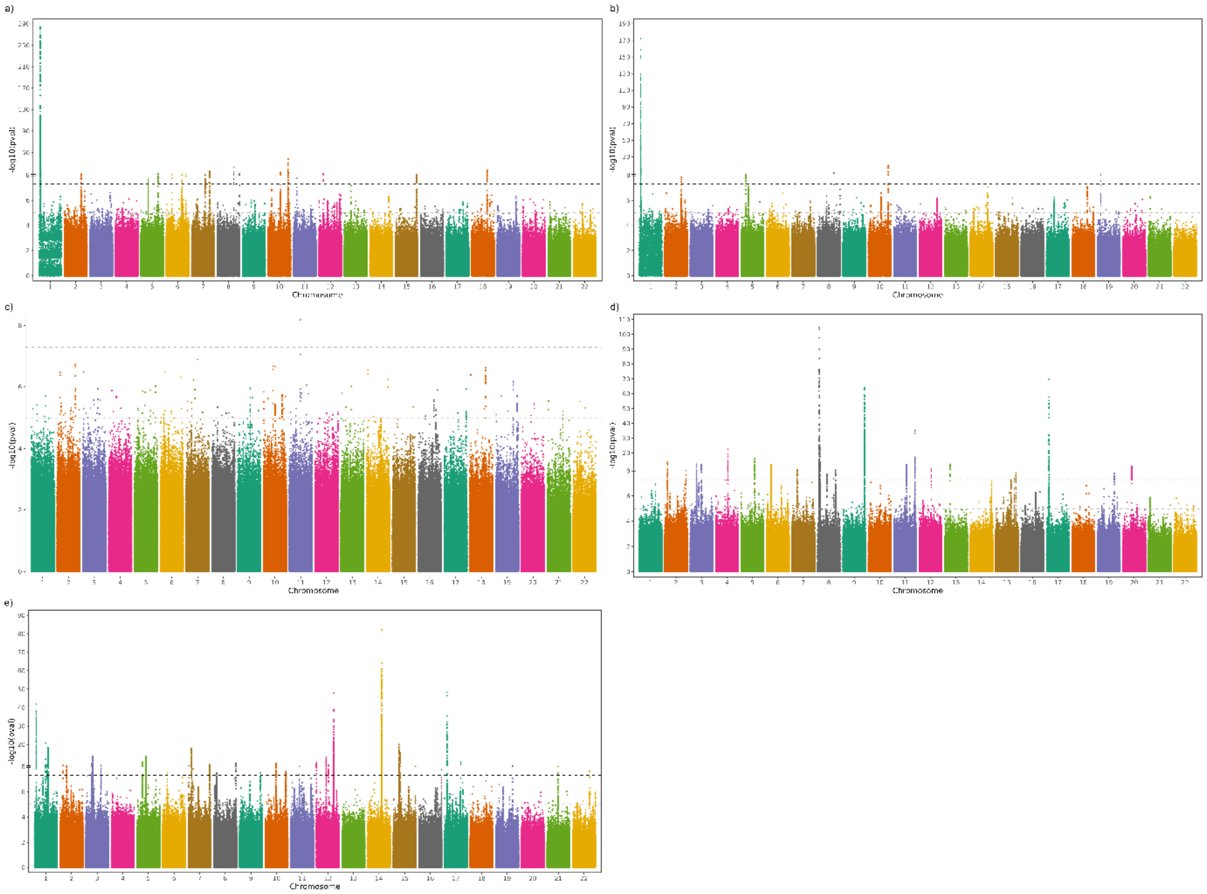

***Figure S2. Manhattan plots of genome-wide association analyses (GWASs) of HCM-associated plasma proteins in UK Biobank.** GWASs of **a)** N-terminal pro-brain natriuretic peptide (NTproBNP) and its precursor **b)** brain natriuretic peptide (NPPB) result in cis-pQTLs at the shared NPPB locus on Chromosome 1 as expected given it encodes the precursor to both these plasma proteins. **c)** Troponin I does not have strong associations and after fine-mapping, there are no genome-wide significant SNPs, hence no genetic instruments. **d)** GWAS of ANGTP2 protein identifies cis-pQTLs at the ANGPT2 locus on chromosome 8 and likewise with GWAS of **e)** LTBP2 protein which identifies cis-pQTLs at the LTBP2 locus on chromosome 18. GWAS analyses were conducted on UKB individuals which did not overlap with the HCM GWAS meta-analysis[34].*

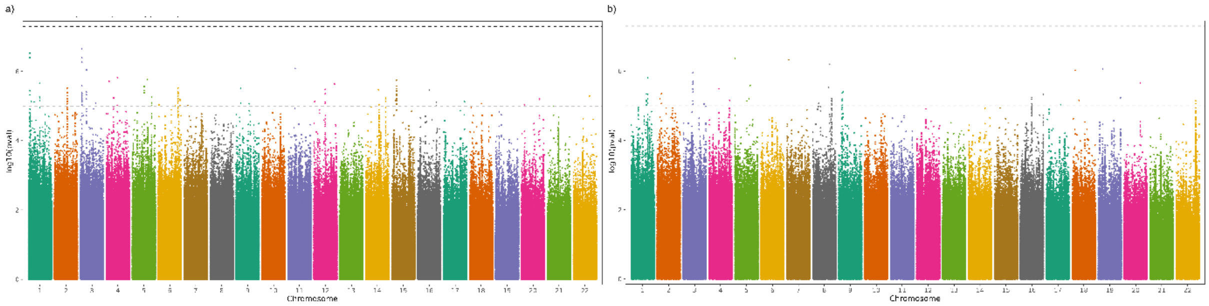

***Figure S3. Manhattan plots of genome-wide association analyses (GWASs) of imaging phenotype-associated plasma proteins in HCM Registry patients. a)** GWAS of N-terminal pro-brain natriuretic peptide (NTproBNP) identifies a cis-pQTL (rs198388) at approximate FDR-significance in the NPPB locus on chromosome 1. **b)** GWAS of Troponin T fails to identify cis-pQTLs but identifies trans-pQTLs mapping to SLC8A1 locus on chromosome 2 (rs72798626) and SNTB1 locus on chromosome 8 (rs11418951).*

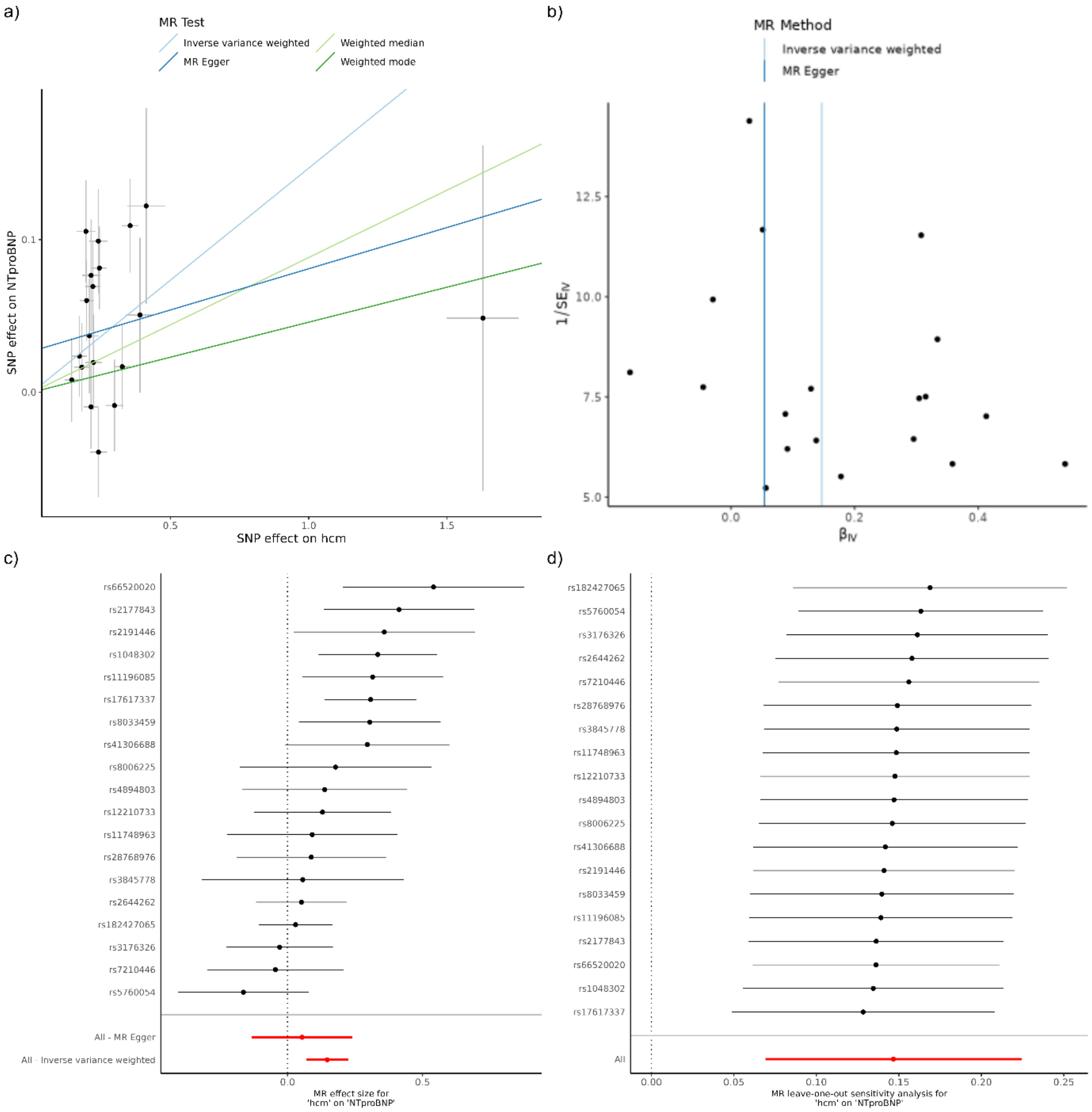

Figure S4. Mendelian randomisation (MR) plots to evaluate the causal relationship between HCM exposure and NTproBNP outcome in HCM Registry. a) *Scatter plot of 19 HCM genetic instruments from the HCMR-less HCM meta-analysis with the respective straight lines reflecting inverse-variance weighted and other robust MR methods.* ***b)*** *Funnel plot showing for each instrument, its precision and effect size to evaluate for directional pleiotropy.* ***c)*** *Single-SNP analyses indicating effect sizes estimates for each genetic instrument.* ***d)*** *Leave-one-out analyses indicating the overall causal estimate in the absence of individual SNPs to assess for outlier-driven effects. Error bars reflect 95% confidence intervals.*

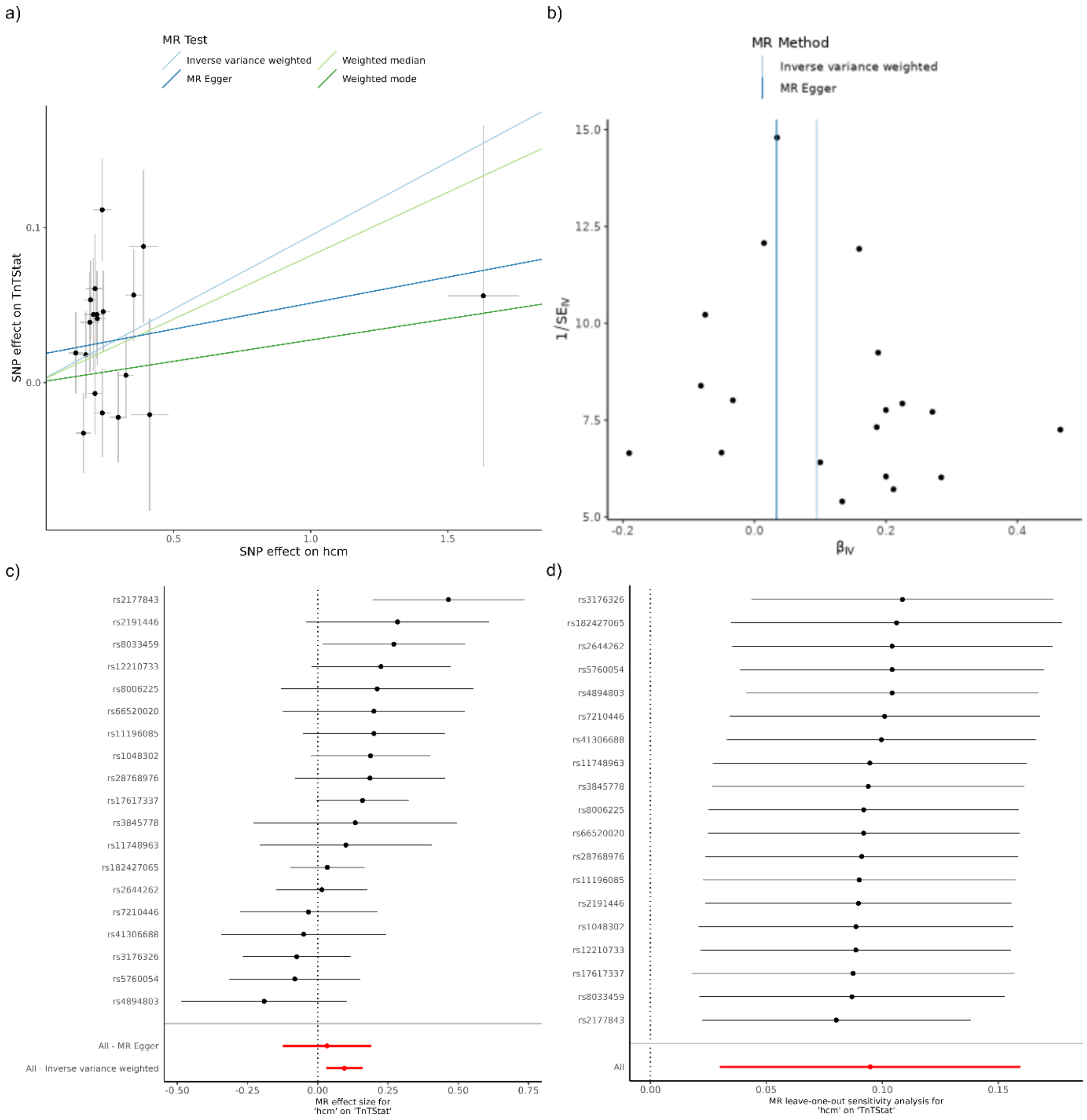

Figure S5. Mendelian randomisation (MR) plots to evaluate the causal relationship between HCM exposure and Troponin T outcome in HCM Registry. a) *Scatter plot of 19 HCM genetic instruments from the HCMR-less HCM meta-analysis with the respective straight lines reflecting inverse-variance weighted and other robust MR methods.* ***b)*** *Funnel plot showing for each instrument, its precision and effect size to evaluate for directional pleiotropy.* ***c)*** *Single-SNP analyses indicating effect sizes estimates for each genetic instrument.* ***d)*** *Leave-one-out analyses indicating the overall causal estimate in the absence of individual SNPs to assess for outlier-driven effects. Error bars reflect 95% confidence intervals.*

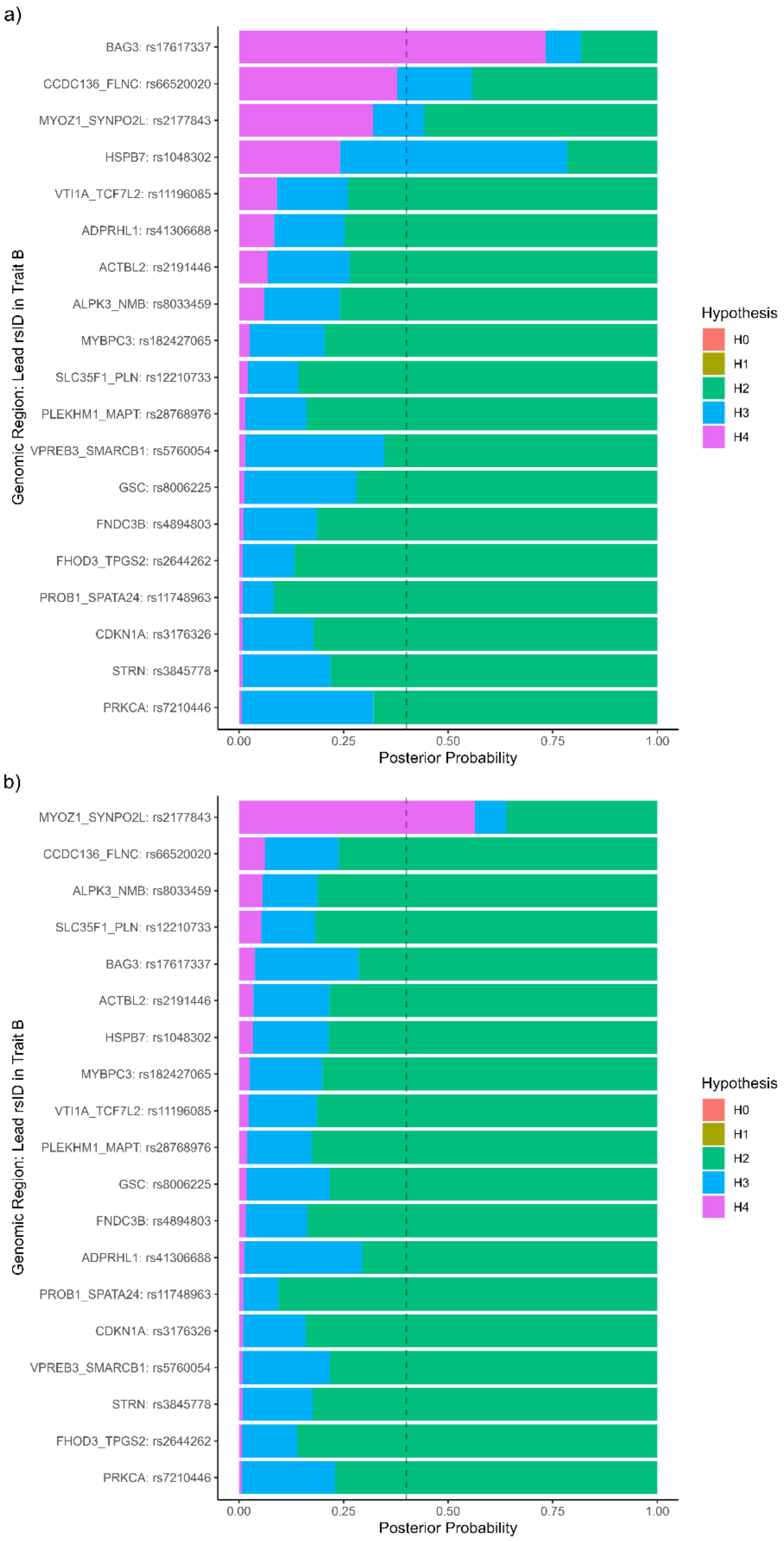

Figure S6. Colocalisation analyses of HCM and plasma protein traits at loci associated with HCM genetic instruments. a) *Colocalisation analysis of HCM instruments' loci with NTproBNP GWAS in HCM Registry indicates substantial evidence for shared associated SNP across both traits in BAG3 locus which may drive its MR signal.* ***b)*** *Colocalisation analysis of HCM instruments' loci with Troponin T GWAS in HCM Registry indicates substantial evidence for shared associated SNP across both traits in MYOZ1/SYNPO2L locus which may drive its MR signal. H0: Hypothesis that associated variants do not exist for either trait, H1: Hypothesis that association with plasma protein trait but not HCM, H2: Hypothesis that association with HCM but not plasma protein, H3: Hypothesis that association with both HCM and plasma protein but via independent SNPs, H4: Hypothesis that association with both HCM and plasma protein but via shared SNP. Dashed line represents posterior probability of H4 = 0.4.*

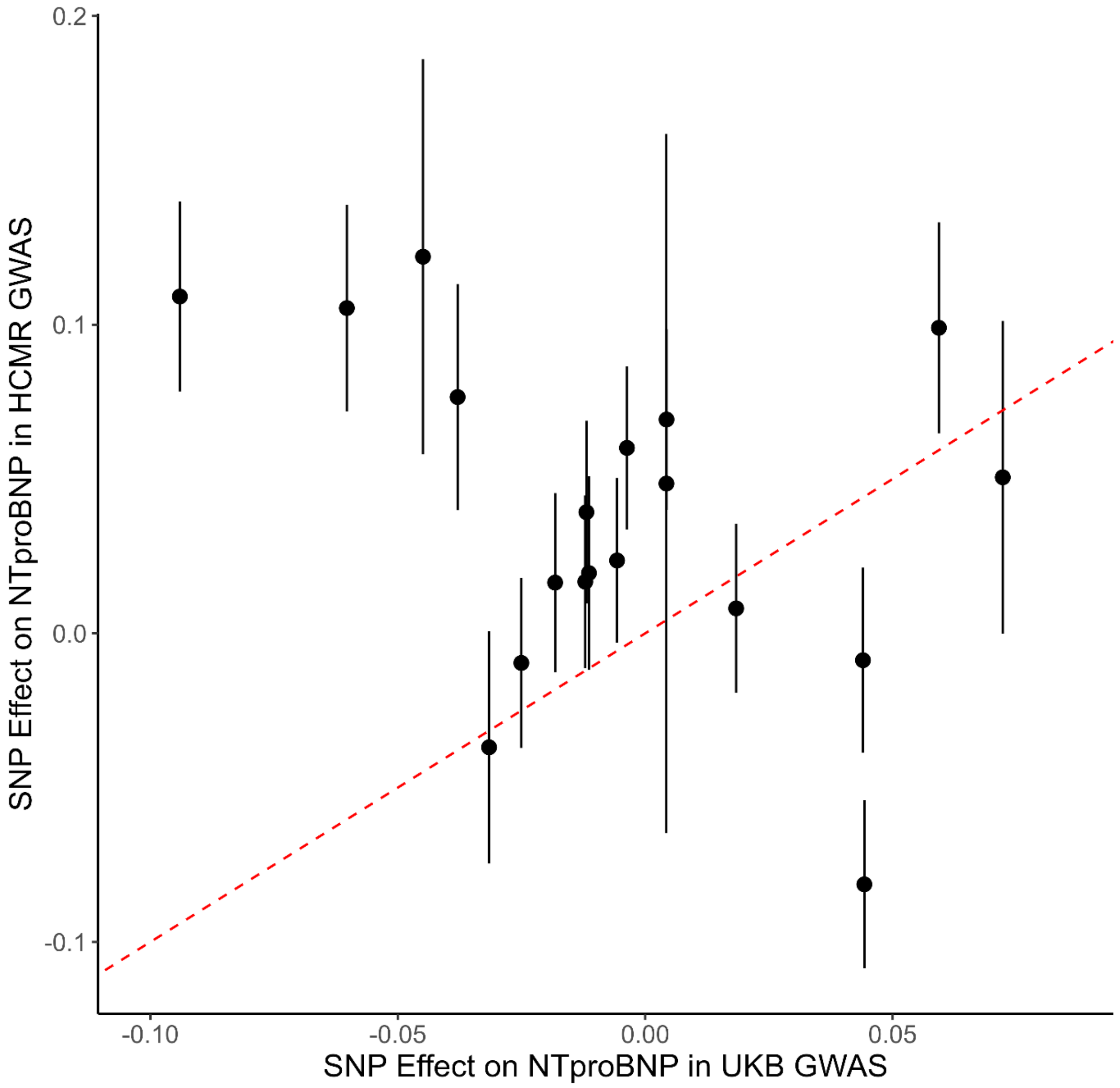

Figure S7. Comparison of HCM genetic instrument effects on NTproBNP in the plasma protein GWASs from UKB compared to in HCMR. *The 19 HCM genetic instruments shared across the Mendelian randomisation analyses for HCM -> NTproBNP in HCMR and HCM -> NTproBNP in UKB are shown here. The red dashed line represents y=x. The greater number of instruments above the equivalence line represents how for the same SNPs have greater beta estimate for NTproBNP in the HCMR-derived GWAS relative to the UKB-derived GWAS. This provides a potential explanation for the greater magnitude of causal estimate for HCM -> NTproBNP in former as opposed to the latter. This may reflect greater severity of HCM cases (and hence elevated NTproBNP levels) in HCMR relative to UKB. Error bars reflect standard error and visual absence of x-axis error bars reflects small standard error of SNP effect estimates in UKB GWAS of NTproBNP.*

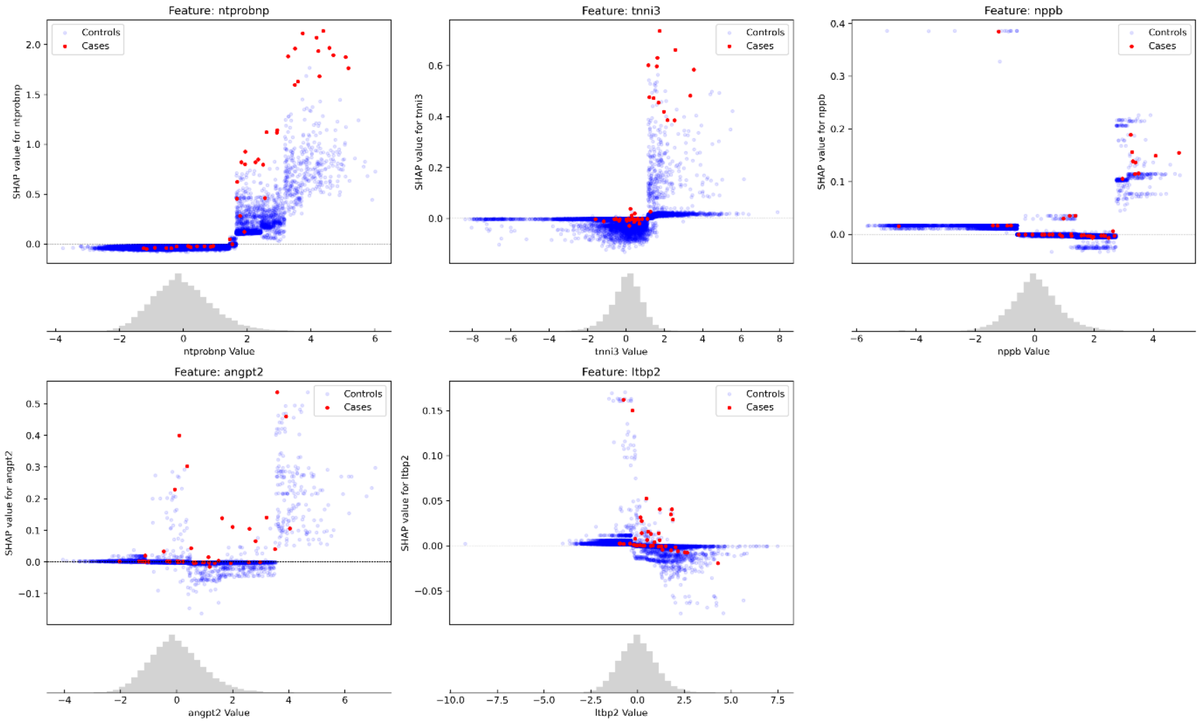

Figure S8. SHAP dependence plots for plasma protein features in trained XGBoost classifier. *Dependence plots compare the SHAP value for each plasma protein (y-axis) against the feature value itself (x-axis) for each individual within the UK Biobank dataset, with prevalent cases (red) and controls (blue) labelled separately. NTproBNP provides a strong indicator of 'case' status in the trained model as protein levels above ~2 SD result in the model assigning substantial 'case' prediction in those individuals (large positive SHAP value). For individuals with levels below that threshold (~2 SD), there is little contribution to model prediction (SHAP value ~ 0). Troponin I (TNNI3) and angiopoietin-2 (ANGPT2) have similar effects whereby large protein values lead to increased case prediction (positive SHAP values) albeit with weaker effects than NTproBNP.*

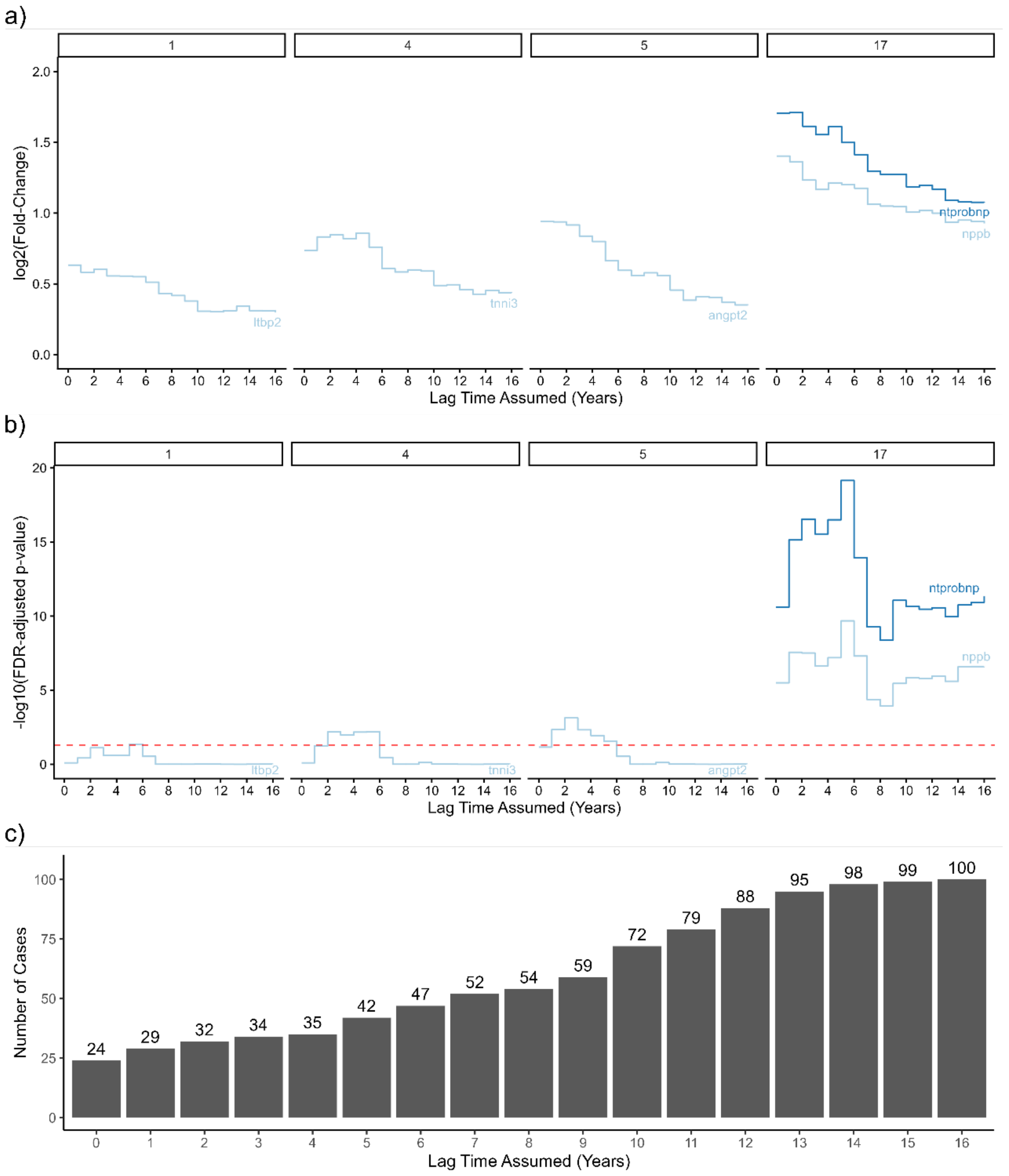

Figure S9. Sensitivity case-control analyses over range of possible assumed lag times between blood sample collection and date of diagnosis in UK Biobank. a) *We assume a potential lag time between true disease manifestation and diagnosis of disease which reflects the potential for individuals to have latent, undiagnosed HCM at blood sample collection date. This lag time ranges from 0 years (i.e. assuming no undiagnosed/latent HCM cases at date of blood sample collection) to 16 years (the maximum possible time from blood sample collection date to dataset cutoff date). Each year instance represents an individual case-control analysis. The y-axis presents the either the* ***a)*** *log2(fold-change) of the plasma protein in the prevalent cases relative to controls in each of these analyses or the* ***b)*** *-log10(adjusted p-value) for that analysis. Known HCM biomarkers (e.g. NTproBNP and Troponin I (TNNI3)) decrease in effect size as the assumed lag time increases which reflects the increasing noise from including cases diagnosed at later dates which may not actually have been cases at blood sample collection. A threshold date of 5 years was selected as a balance between increased statistical power for downstream analyses (by increasing the number of cases) and minimising such noise (as shown by known biomarkers' effects not substantially changing from 0 to 5 years instances). Plasma proteins are plotted if they are 5% FDR-significant in at least one of the assumed lag times. Plot is facetted to separate plasma proteins by the number of lag time instances (maximum 17) for which the plasma protein is FDR-significant.* ***c)*** *Number of individuals considered as prevalent 'cases' as the assumed lag time increases from 0 years (i.e. no latent, undiagnosed HCM cases at date of blood sample collection) to 16 years. This represents an increase in statistical power (including more potentially undiagnosed cases) as you increase the assumed lag time.*

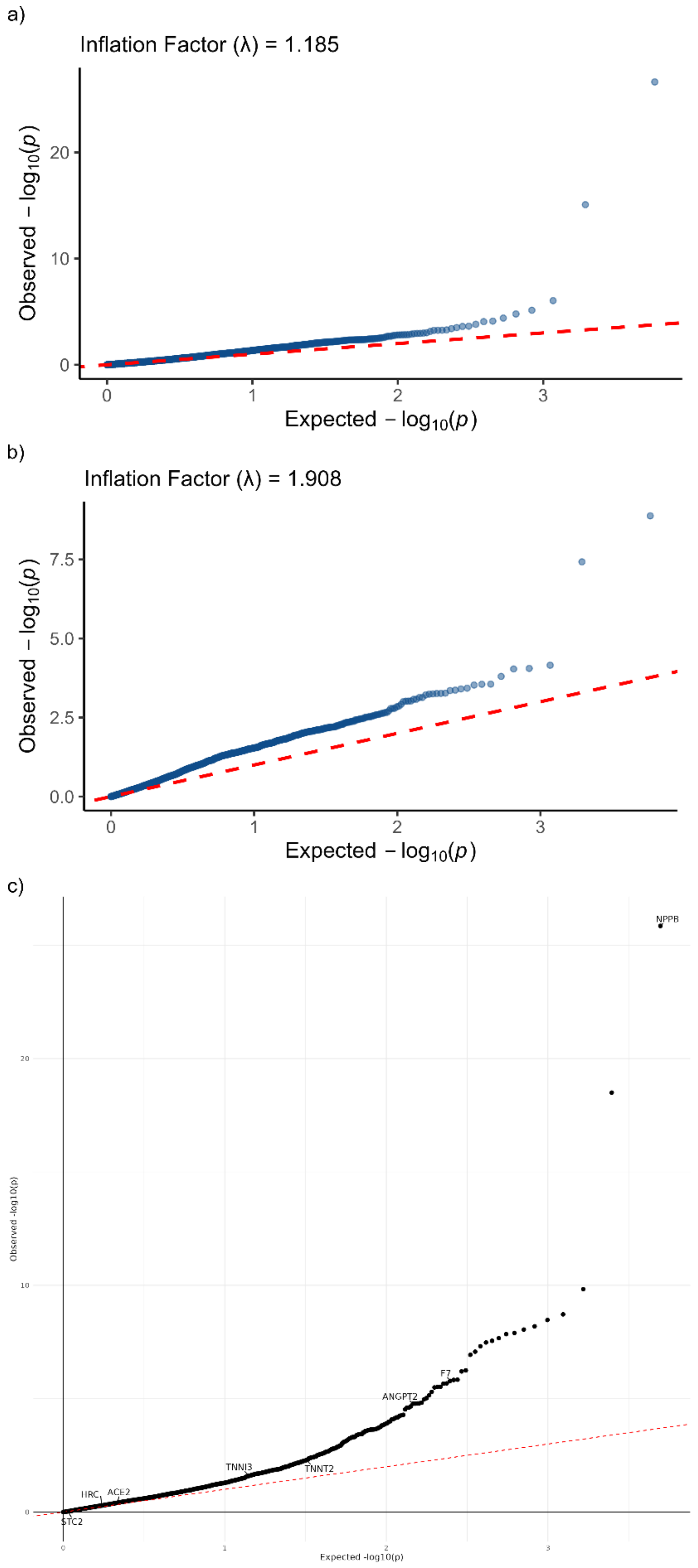

Figure S10. **Quantile-quantile plots for discovery association analyses in UK Biobank and DeCODE Genetics datasets.** These plots demonstrate inflation in the test statistics from analyses in the UK Biobank (**a)** differential expression analyses and **b)** time-to-diagnosis analyses) and ***c)*** deCODE Genetics case-control differential expression datasets, necessitating the need for test statistic adjustment via the lambda inflation factor.
